# Global comparative transcriptome analysis of cartilage formation *in vivo*

**DOI:** 10.1186/1471-213X-9-20

**Published:** 2009-03-10

**Authors:** Trevor L Cameron, Daniele Belluoccio, Peter G Farlie, Bent Brachvogel, John F Bateman

**Affiliations:** 1 Murdoch Childrens Research Institute and Department of Paediatrics, University of Melbourne, Royal Children's Hospital, Parkville, Victoria 3052, Australia; 2 Center for Biochemistry, Medical Faculty, University of Cologne, Germany

## Abstract

**Background:**

During vertebrate embryogenesis the initial stages of bone formation by endochondral ossification involve the aggregation and proliferation of mesenchymal cells into condensations. Continued growth of the condensations and differentiation of the mesenchymal cells into chondrocytes results in the formation of cartilage templates, or anlagen, which prefigure the shape of the future bones. The chondrocytes in the anlagen further differentiate by undergoing a complex sequence of maturation and hypertrophy, and are eventually replaced by mineralized bone. Regulation of the onset of chondrogenesis is incompletely understood, and would be informed by comprehensive analyses of *in vivo *gene expression.

**Results:**

Tibial and fibular pre-condensed mesenchyme was microdissected from mouse hind limbs at 11.5 dpc, and the corresponding condensations at 12.5 dpc and cartilage anlagen at 13.5 dpc. Total RNA was isolated, and cRNA generated by linear amplification was interrogated using mouse whole genome microarrays. Differential expression was validated by quantitative PCR for *Agc1*, *Bmp8a*, *Col2a1*, *Fgfr4*, *Foxa3*, *Gdf5*, *Klf2*, *Klf4*, *Lepre1*, *Ncad*, *Sox11*, and *Trpv4*. Further, independent validation of the microarray data was achieved by *in situ *hybridization to analyse the expression of *Lepre1*, *Pcdh8*, *Sox11*, and *Trpv4 *from 11.5 dpc to 13.5 dpc during mouse hind limb development. We found significant differential expression of 931 genes during these early stages of chondrogenesis. Of these, 380 genes were down-regulated and 551 up-regulated. Our studies characterized the expression pattern of gene families previously associated with chondrogenesis, such as adhesion molecules, secreted signalling molecules, transcription factors, and extracellular matrix components. Gene ontology approaches identified 892 differentially expressed genes not previously identified during the initiation of chondrogenesis. These included several *Bmp, Gdf, Wnt, Sox and Fox *family members.

**Conclusion:**

These data represent the first global gene expression profiling analysis of chondrogenic tissues during *in vivo *development. They identify genes for further study on their functional roles in chondrogenesis, and provide a comprehensive and important resource for future studies on cartilage development and disease.

## Background

Developing a detailed knowledge of the developmental pathways involved in limb skeletogenesis is important for understanding skeletal abnormalities and disease processes, and for further unravelling the fundamental regulatory pathways that control development. The development of the vertebrate limb skeleton is initiated when multipotent mesenchymal cells in the limb bud aggregate to form mesenchymal condensations which prefigure the skeletal elements. Cells within the pre-chondrogenic condensation up-regulate cell adhesion mechanisms and begin to synthesize specific extracellular matrix molecules, and the condensations expand through a combination of proliferation and recruitment of surrounding mesenchyme. The generation of these condensations creates an environment which is conducive to chondrogenic differentiation [1,2]. As the cells differentiate into chondrocytes, they synthesize a framework of cartilage matrix, known as an anlage, in the approximate shape of the future bone. Chondrocytes in the centre of the anlage proceed through a series of discrete developmental stages that include proliferation, maturation and hypertrophy [1,3-7]. The hypertrophic cartilage is first calcified and then, following vascular invasion, replaced by primary bone that is subsequently remodelled to form secondary bone. This process radiates outwards from the centre of the anlage with the development of highly ordered growth plates that separate the cartilaginous epiphyses from the bony diaphysis. Later in development, secondary centres of ossification develop within the epiphyses and, with subsequent fusion of the ossification centres during puberty, endochondral ossification and bone growth ceases.

The regulatory network that controls early chondrogenesis is incompletely understood although several key components have been characterized. Prior to pre-chondrogenic mesenchymal condensation in the developing vertebrate limb bud, Bmp expression is upregulated in the mesenchyme flanking the anterior and posterior margins and in the presumptive interdigital mesenchyme, in a manner consistent with a role for these genes in limb patterning and in initiating chondrogenesis and skeletal development [8,9]. Furthermore, viral misexpression of the Bmp-antagonist, Noggin, in the developing limb during embryogenesis [10] and targeted over-expression of Noggin in chondroprogenitor cells [11], both result in complete blockage of mesenchymal condensation and chondrogenesis, proving the requirement for Bmp signalling in the initiation of chondrogenesis. Bmp signalling serves multiple purposes during the initiation of chondrogenesis; it may contribute to the recruitment of cells to the condensation, the proliferation of cells within the condensation, as well as regulating the expression of genes involved in driving the differentiation of condensed mesenchyme [2]. In addition to the roles of Bmps in skeletal development *per se*, Bmp, along with Bmp antagonists (eg., chordin and noggin) and members of the Gdf (growth and differentiation factor) family are involved in controlling the cartilage condensations that develop into the synovial joint. The Fgf family of growth factors and their receptors also play an important role in chondrocyte differentiation, possibly by limiting chondrocyte proliferation, since activating mutations in *FGFR3 *cause achondroplasia and thanatophoric dysplasia and *Fgfr3*-deficient mice have an enlarged growth plate. A critical component of chondrocyte lineage specification is Sox9 (a high mobility group transcription factor), which exerts its influence over chondrogenesis, in part, by regulating the expression of multiple cartilage-specific proteins including Col2a1 [12]. The influence of Sox9 on chondrogenesis is largely dependent upon Sox5 and Sox6, with which it co-regulates the chondrogenic program [13].

In addition to the signalling cascades, the extracellular matrix (ECM) plays a fundamental role in morphogenesis and development by regulating cell differentiation, proliferation, adhesion, and migration, and by modulating growth factor bioavailability. Mesenchymal condensations are characterized by the expression of a specific ECM, components of which include fibronectin [14], tenascin C [15], NG2 proteoglycan [16], syndecan [17], and chondroitin-sulphate proteoglycans [18]. The ECM has important roles in promoting the condensation and differentiation of mesenchymal cells [2]. As the cells differentiate into chondrocytes they express specific ECM components with unique functional and biological characteristics which are critical for cartilage structure and function [19,20]. The predominant, well-characterized cartilage matrix components are the proteoglycan, aggrecan, and members of the collagen protein family. Collagen II is a major homotrimeric collagen expressed throughout the cartilage that interacts specifically with other minor collagens such as IX and XI to modulate interfibrillar interactions between the heterotypic collagen fibrils [21]. Mutations in many of these collagen genes have been identified in human chondrodysplasias and in mouse models [7,22,23]. Collagen VI is a microfibrillar collagen which is found localized in the pericellular space around chondrocytes in epiphyseal cartilage.

Cartilage contains many other proteins, some of which have been well-characterized and whose roles in cartilage structure and function have been elucidated [19,24-27]. Some of the best characterized components are the small leucine-rich proteins (SLRPs), such as decorin, fibromodulin, lumican and biglycan which interact with collagen fibrils and influence collagen fibrillar architecture and function. These SLRPs have growth factor binding properties that may have profound influences on cartilage cell proliferation. Cartilage oligomeric matrix protein also binds collagen fibrils and may also mediate cell-matrix interactions. Other proteins include chondroadherin and thrombospondin which have cell-matrix binding properties; tenascins, osteonectin and members of the matrilin family, matrilins 1 and 3, that are thought to form interacting assemblies integrating the collagen and proteoglycan networks.

While a number of the chondrogenesis genes have been extensively characterized, the full spectrum of components involved in regulating chondrogenesis during limb development has yet to be determined. However, with the advent of high quality whole genome microarrays we now have the unique opportunity to determine the comprehensive gene expression pattern of processes such as chondrogenesis, to define novel components and further unravel the complex developmental processes involved. The application of such expression profiling approaches to cartilage formation *in vivo *has thus far been restricted by the technical challenge of obtaining high quality RNA from the target tissues. Consequently, expression studies to date have been conducted on either whole mouse limbs [28,29] or on *in vitro *models of chondrogenesis [30-32]. While these studies have been useful in identifying some chondrogenic genes, they suffer from the limitations of analysing heterogeneous tissues or the complex gene expression consequences that may result from *in vitro *culture.

In this study we report the first global gene expression profiling analysis of the transition of pre-condensed mesenchymal cells into mesenchymal condensations, and their subsequent differentiation into chondrocytes, using tissue microdissected from *in vivo *mouse limb buds. By comparison of the gene expression profiles of cartilage pre-condensation tissue at 11.5 dpc, mesenchymal condensations at 12.5 dpc, and differentiated cartilage tissue at 13.5 dpc, we were able to identify the candidate gene cohorts involved in the initiation of chondrogenesis and in the early development of cartilage anlagen. As well as identifying novel genes that may be critical in the regulation and maintenance of chondrogenesis, our data allowed us to present the first comprehensive expression analysis of the known developmentally important gene families during *in vivo *chondrogenesis during mouse limb skeletal development. These data provide confirmation of the role of many members of these key gene families, and also implicate other members of these, and other gene families in chondrogenesis for the first time.

## Results and discussion

### Microdissected samples from 11.5 dpc to 13.5 dpc encompass the initial developmental steps of chondrogenesis

**Figure 1:**
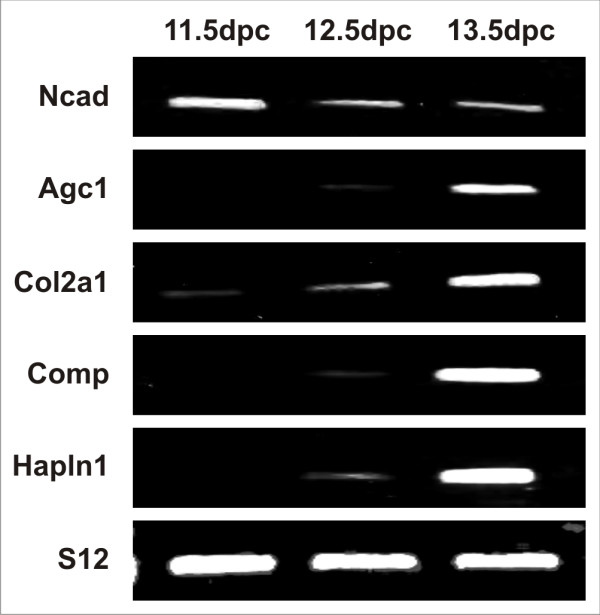
**Semi-quantitative PCR analysis of cartilage specific gene expression in microdissected limb cartilage condensations**. Agarose gel electrophoresis of PCR products generated by primers specific for N-cadherin (*Ncad*), aggrecan (*Agc1*), procollagen II alpha I (*Col2a1*), cartilage link protein (*Hapln1*; hyaluronan and proteoglycan link protein 1), and ribosomal protein S12 (*S12*).

Prior to microarray analysis, the differential expression of selected key markers of mesenchymal condensation and chondrogenesis was assayed by semi-quantitative PCR to confirm the developmental stage represented by each microdissected tissue sample (Fig. 1). The genes chosen for semi-quantitative PCR analysis were N-cadherin (*Ncad*), aggrecan (*Agc1*), collagen II (*Col2a1*), cartilage oligomeric matrix protein (*Comp*), cartilage link protein (*Hapln1*, hyaluronan and proteoglycan link protein 1) and the gene encoding S12 ribosomal RNA, which was included as a loading standard. Ncad is a cell-cell adhesion molecule whose role in mesenchymal condensation during limb development has been well characterized [33]. Expression of aggrecan, collagen II, Comp and cartilage link protein is indicative of cartilage formation [34].

Semi-quantitative PCR demonstrated *Ncad *transcripts were abundant at 11.5 dpc, immediately prior to the commencement of mesenchymal condensation. A marked decrease in *Ncad *transcription was observed by 12.5 dpc and this low level of expression was maintained until 13.5 dpc. *Agc1 *and *Col2a1 *were present at low levels in 11.5 dpc embryos, well before the deposition of significant extracellular matrix. Transcription of both these markers increased by 12.5 dpc, and they became highly abundant by 13.5 dpc. Neither *Comp *nor *Hapln1 *transcripts were present at levels detectable by semi-quantitative RT-PCR at 11.5 dpc. Low-level transcription of *Comp *and *Hapln1 *was observed at 12.5 dpc, and increased dramatically by 13.5 dpc. These data provided clear evidence that the microdissected regions of the limb buds represented mesenchymal condensations undergoing the *in vivo *transition from undifferentiated chondroprogenitor cells to differentiated chondrocytes.

### Whole genome expression profiling

**Figure 2:**
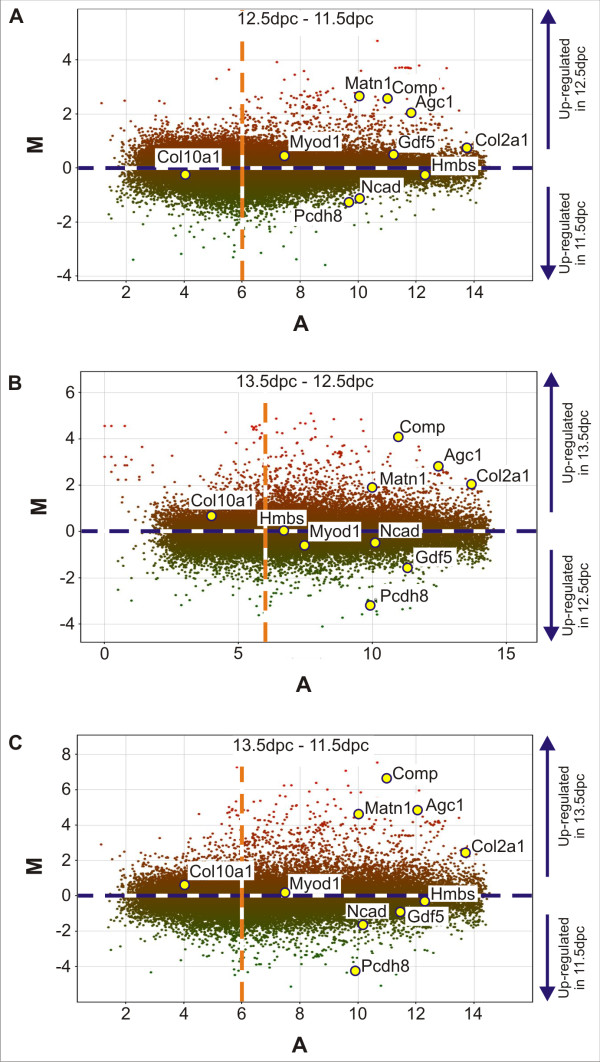
**Mouse whole genome microarray M/A scatter plots**. **A) **12.5 dpc – 11.5 dpc. **B) **13.5 dpc – 12.5 dpc. **C) **13.5 dpc – 11.5 dpc. M represents the log ratio of the two dyes and A represents the average Log_2 _intensity. Yellow spots with blue outline indicate the expression data for selected genes. The dashed blue line indicates a fold difference of zero representing no differential expression between the two samples analysed. The dashed orange line indicates a average Log_2 _intensity (A) of 6, representing the background signal intensity.

As an overall representation of the microarray hybridizations, M/A scatter plots were generated for each time point comparison in which the mean signal intensity (Average Log_2 _Intensity; A) for each microarray probe was plotted against the relative fold difference for that probe (Fig. 2). Negative control probes, which are not complementary to any mouse mRNA sequences, have been included on these microarrays by the manufacturer to gauge non-specific, background fluorescence. Using these probes, it was determined that an average Log_2 _intensity of less than or equal to six represented background fluorescence (indicated by dashed orange line in Fig. 2).

Scatter plot comparisons were generated by subtracting the signal intensity of 11.5 dpc from 12.5 dpc (Fig. 2A), 12.5 dpc from 13.5 dpc (Fig. 2B), and 11.5 dpc from 13.5 dpc (Fig. 2C). By plotting the data in this manner for each of the three comparisons, genes which were expressed in equal relative abundance between time points were plotted close to zero on the relative fold difference (vertical; M) axis. Genes which were more highly expressed at the later time points were plotted on the positive scale of the relative fold difference axis (red). Conversely, genes which were more highly expressed at the earlier time points were plotted on the negative scale of the relative fold difference axis (green). The data-points for selected markers of mesenchymal condensation (*Ncad*, growth differentiation factor 5 – *Gdf5*, and protocadherin 8 – *Pcdh8*), chondrogenesis (*Agc1*, *Col2a1*, *Comp *and matrilin 1 – *Matn1*), myogenesis (myogenic differentiation 1 – *Myod1*), and chondrocyte hypertrophy (procollagen type 10 alpha 1 – *Col10a1*), as well as a house keeping gene as an internal standard (hydroxymethylbilane synthase – *Hmbs*) have been highlighted in each scatter plot. It can be seen in each of the plots in Figure 2 that the selected markers of chondrogenesis were found to be expressed more highly in the later time point, while markers of mesenchymal condensation were found to be expressed more highly at the earlier time point. Consistently, the average Log_2 _intensity for *Col10a1 *was found to be below six, confirming that *Col10a1*, a marker of cartilage hypertrophy, was not expressed in any of the microdissected tissues and that the cartilage anlagen microdissected at 13.5 dpc were pre-hypertrophic. For *Myod1*, the average Log_2 _intensity was found to be very close to background level and the relative fold difference close to zero, indicating negligible *Myod1 *expression in these tissues, and suggesting that there was no significant contamination of the microdissected tissues by myogenic precursors.

In order to generate global representations of biological processes driving chondrogenesis, the microarray data were mined using OntoExpress gene ontology software. Genes up-regulated or down-regulated between 11.5 dpc to 13.5 dpc by at least three-fold, and for which an average Log_2 _intensity >6 was detected, were classified according to biological process (see Additional file 1). By this method, 931 significantly differentially expressed genes were identified – 380 down-regulated genes and 551 up-regulated genes. These genes are shown in Additional file 2. The full microarray dataset are available from the Gene Expression Omnibus (GEO) database repository .

### Validation of microarray data

Three approaches were taken to validate the microarray dataset generated in the present study – quantitative PCR (qPCR), *in situ *hybridization, and an extensive comparison of the gene expression dataset generated in this study with expression and function data pertaining to the same genes in the scientific literature. To provide microarray validation over a wide range of gene expression levels we performed qPCR on genes expressed at high levels by chondrocytes, such as extracellular matrix components (eg. *Col2a1*), and also those expressed at much lower levels, such as transcription factors (eg. *Foxa3*). In addition, array validation was performed for representative genes from the developmentally important gene families; adhesion molecules, secreted signalling molecules, transcription factors and extracellular matrix molecules.

### Quantitative PCR

**Figure 3:**
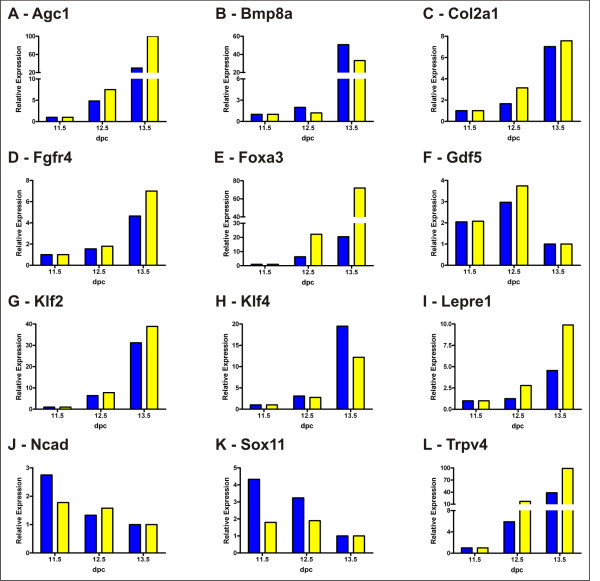
**Comparison of differential expression profiles of selected genes analysed by quantitative real time PCR or microarrays**. Expression was analyzed in amplified mRNA by quantitative real time PCR analysis (qPCR; yellow bars) or microarray analysis (blue bars) for **A) **aggrecan (*Agc1*); **B)**, *Bmp8a*; C), *Col2a1*; D), *Fgfr4*; E), *Foxa3*, F), *Gdf5*; G), *Klf2*, H), *Klf4*; I), *Lepre1*; J), *Ncad*; K), *Sox11*; L) *Trpv4*. qPCR experiments were performed in triplicate.

Using unmodified cRNA samples amplified in parallel to those cRNA samples generated for the microarray analyses, qPCR was performed on selected genes as a technical validation for the microarray data. qPCR was performed on *Agc1 *(Fig. 3A), *Bmp8a *(Fig. 3B), *Col2a1 *(Fig. 3C), *Fgfr4 *(Fig. 3D), *Foxa3 *(Fig. 3E), *Gdf5 *(Fig. 3F), *Klf2 *(Fig. 3G), *Klf4 *(Fig. 3H), *Lepre1 *(Fig. 3I), *Ncad *(Fig. 3J), *Sox11 *(Fig. 3K), and *Trpv4 *(Fig. 3L). For each marker, a close correlation was observed between the pattern of differential gene expression determined using either technique, with highest expression for markers of mesenchymal condensation seen between 11.5 dpc and 12.5 dpc, and highest expression for markers of chondrogenesis seen at 13.5 dpc.

### *In situ *hybridization

**Figure 4:**
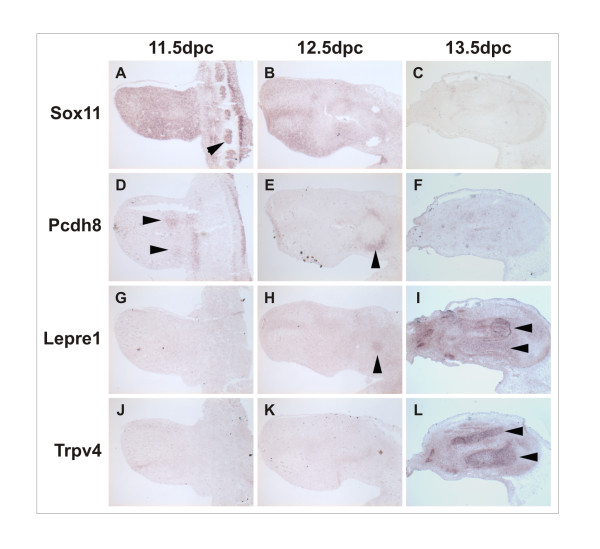
**In situ hybridization analysis of Sox11, Pcdh8, Lepre1 and Trpv4 in embryonic mouse hindlimbs**. Antisense probes were used to determine the level and distribution of mRNA for each gene. Expression of Sox11 is widespread throughout the limb bud and somites (arrowhead) at E11.5 but declines in level by E12.5 and becomes undetectable by E13.5. Pcdh8 is expressed within the pre-condensations of the tibia and fibula (arrowheads) at E11.5 but is downregulated by the condensation phase at E12.5. At this stage expression is detected in the tissue surrounding the femoral condensation (arrowhead) but declines to background levels by E13.5. Leprecan1 is not detectable at E11.5 but becomes visible in the femoral condensation at E12.5 (arrowhead) and is upregulated within the tibia and fibula at E13.5 (arrowheads). Trpv4 is not detectable within the limb buds at E11.5 or E12.5 but is strongly upregulated in the tibia and fibula at E13.5 (arrowheads). Distal is to the left in all images.

In order to validate the microarray data further with independent, biological replicates, *in situ *hybridization was performed on sagittal sections from 11.5 dpc, 12.5 dpc, and 13.5 dpc mouse hind limb buds using probes for *Sox11 *(Fig. 4A–C), *Pcdh8 *(Fig. 4D–F), *Lepre1 *(Fig. 4G–I), and *Trpv4 *(Fig. 4J–L). In each case, the gene expression detected by *in situ *hybridization corroborated the expression profiles generated by microarray and clarified the expression of these genes in the limb bud beyond the microdissected areas.

Strong expression of *Sox11 *was observed throughout the limb bud and also in the adjacent somites at 11.5 dpc (Fig. 4A). At 12.5 dpc, expression of *Sox11 *was generally weaker than at 11.5 dpc, and found to be most highly expressed in the interdigital mesenchyme distally, and localized to the tibial and fibular mesenchymal condensations, as well as the anterior and posterior margins of the limb bud proximally (Fig. 4B). At 13.5 dpc, *Sox11 *expression was very weak distally and absent from the tibia and fibula (Fig. 4C). This pattern of *Sox11 *expression agrees with the expression profiles generated by microarray and qPCR, where *Sox11 *was found to be down-regulated during development of the tibial and fibular cartilage anlagen. Similarly, *Pcdh8 *also exhibited declining expression levels from 11.5 dpc–13.5 dpc. At 11.5 dpc, *Pcdh8 *expression was detected in the precondensed tibial and fibular mesenchyme (Fig. 4D) but was absent from the distal limb at E12.5 (Fig 4E). In contrast, Pcdh8 expression at E12.5 was predominantly detected surrounding the femoral condensation (Fig 4E) but became undetectable within the limb by E13.5 (Fig 4F). Thus, as with the expression profile generated by microarray for *Pcdh8*, *in situ *hybridization confirmed that expression of *Pcdh8 *is down-regulated in the areas of tibial and fibular development.

*Lepre1 *expression was not detectable in the limb bud at 11.5 dpc (Fig. 4G). By 12.5 dpc, weak expression of *Lepre1 *was observed in the femoral, tibial, and fibular prechondrogenic mesenchymal condensations (Fig. 4H). *Lepre1 *was found to be highly expressed throughout the cartilage anlagen, though not in the perichondrium, at 13.5 dpc (Fig 4I). Similar results were obtained for *Trpv4*, which was not detectable at either 11.5 dpc (Fig. 4J) or 12.5 dpc (Fig. 4K), but which was highly expressed throughout the cartilage anlagen at 13.5 dpc (Fig. 4L).

The *in situ *hybridization data presented here for *Sox11*, *Pcdh8*, *Lepre1*, and *Trpv4 *are consistent with the expression profiles generated for these genes in the present study by microarray analysis and qPCR. In so doing, they reinforce the utility of the present microarray dataset for providing accurate information about the expression profiles of developing chondrogenic tissues *in vivo*.

### Comparative literature analysis

As a third approach to validating the microarray data generated in the present study, an extensive survey of the scientific literature was carried out in order to compare our gene expression dataset with expression and function data pertaining to the same genes examined in previous studies (see Additional file 3). For the 50 genes surveyed, we found that our expression profiles were highly consistent with previously published data, emphasising the value of the present dataset as a tool for further understanding the dynamic gene expression environment controlling *in vivo *chondrogenesis.

### Differential expression of gene families during chondrogenesis

#### Adhesion Molecules

**Figure 5:**
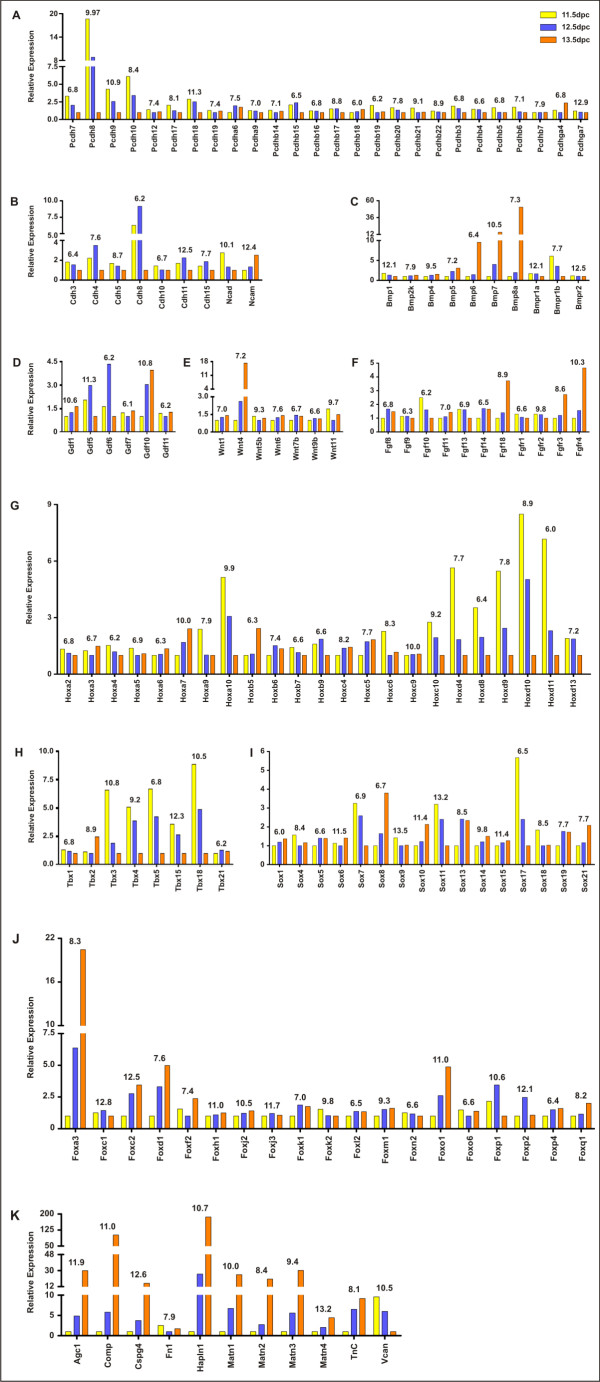
**Microarray differential expression profiles of selected developmental gene families**. **A)**, protocadherins (*Pcdh*); **B)**, cadherins (*Cdh*); **C)**, bone morphogenetic proteins and bone morphogenetic protein receptors (*Bmp *and *Bmpr*); **D)**, growth differentiation factors (*Gdf*); **E)**, Wnt family, (*Wnt*); **F)**, fibroblast growth factors and fibroblast growth factor receptors (*Fgf *and *Fgfr*); **G)**, homeobox transcription factors (*Hox*); **H)**, T-box transcription factors (*Tbx*); **I)**, Sry-like homeobox transcription factors (*Sox*); **J)**, forkhead/winged helix box transcription factors (*Fox*); **K)**, selected non-collagenous extracellular matrix molecules. Yellow bars indicate relative expression at 11.5 dpc. Blue bars indicate relative expression at 12.5 dpc. Orange bars indicate relative expression at 13.5 dpc. For each gene, the time point at which the relative expression was lowest was defined as one, and this was used to calculate the relative expression at the remaining time points. Numbers above each gene denote average Log_2 _intensity (A) for that gene.

Consistent with their roles in mediating cell-cell contacts, it was found that most protocadherins (Fig. 5A) and cadherins (Fig. 5B) were down-regulated from 11.5 dpc – immediately prior to the commencement of mesenchymal condensation, to 13.5 dpc – when cartilage anlagen have formed. The protocadherins are a family of type I membrane proteins belonging to the cadherin superfamily which are characterized by multiple extracellular cadherin domains and unique intracellular domains bridged by a single membrane-spanning segment [35]. The function of the protocadherins is unclear and while they may function in cell-cell adhesion, they are also likely to have a role in intercellular signalling. Of the differentially regulated protocadherin (*Pcdh*) genes (Fig. 5A), the most highly down-regulated were *Pcdh8 *and *Pcdh10*. *Pcdh9 *and *Pcdh18 *were also found to be significantly down-regulated, but more highly expressed than *Pcdh8 *and *Pcdh10*. None of the protocadherins were found to be significantly up-regulated during *in vivo *chondrogenesis. To date, none of the protocadherins have been implicated in chondrogenesis, and *Pcdh8 *is the only protocadherin previously found to be expressed during limb development [36].

The cadherin genes encode membrane-spanning proteins characterized by multiple cadherin domains which mediate calcium-dependent homophilic adhesion between neighbouring cells in a wide range of developmental processes [37]. Among the cadherin (*Cdh*) genes (Fig. 5B), *Cdh8 *and *Cdh4 *were found to exhibit the highest differential expression. Notably, expression of both of these genes peaked during mesenchymal condensation at 12.5 dpc, before being down-regulated in the cartilage anlagen at 13.5 dpc. *Cdh11*, although not significantly differentially expressed, was found to be very highly expressed throughout *in vivo *chondrogenesis. *Cdh11 *has recently been shown to be a discriminative factor between articular and growth plate cartilage chondrocytes [38]. N-cadherin (*Ncad*, *Cdh2*) was found to be very highly expressed and was down-regulated by almost 3-fold from 11.5 dpc to 13.5 dpc, consistent with its characterized role in condensation.

*Ncam *was highly expressed throughout *in vivo *chondrogenesis, and marginally up-regulated between 11.5 dpc and 13.5 dpc. It is recognized that in the context of chondrogenesis, cell-cell adhesion is most likely mediated primarily by Cdh2 [33] and Cdh11 – in conjunction with other factors such as Ncam1 [39]. This view is supported by the findings presented here, in which *Ncam1*, along with *Cdh2 *and *Cdh11 *were highly expressed during the initiation of chondrogenesis, while other cadherins were expressed at a much lower level (Fig. 5B).

#### Secreted signalling molecules

The relative differential expression of genes belonging to major families of signalling molecules secreted during *in vivo *chondrogenesis are shown (Fig. 5C–F). Included in this category are the bone morphogenetic protein (*Bmp*) family (Fig. 5C), the growth differentiation factor (*Gdf*) family (Fig. 5D), the wingless-related MMTV integration site (*Wnt*) family (Fig. 5E), and the fibroblast growth factor family (Fig. 5F). The chondroinductive potential of the Bmp family has been extensively analysed, and it is known that chondrogenesis may be induced by the activity of Bmp2 [40], Bmp4 [41], Bmp5 [42], Bmp6 [43], and Bmp7 [44]. Moreover, expression of Bmp8a in developing long bones has been demonstrated, and it is known to map to the mouse achondroplasia locus [45], suggesting that it too may be an important regulator of chondrogenesis. Consistent with these observations, *Bmp8a *was found to be the most highly differentially expressed Bmp gene, up-regulated over 50-fold between 11.5 dpc and 13.5 dpc. *Bmp6 *and *Bmp7 *were also highly differentially expressed. *Bmp7 *was more highly expressed than *Bmp8a*, while overall expression of *Bmp6 *was low. Although not highly differentially expressed, *Bmp1 *was found, like *Bmp7*, to be very highly expressed. Expression of *Bmp2 *was found to be absent from this assay of *in vivo *chondrogenesis.

It is known that the Gdf family contributes to the regulation of chondrogenesis. Gdf5 may induce chondrogenesis *in vitro *[46], and it has been used to induce ectopic chondrogenesis *in vivo*, in part by stimulating expression of another marker of chondrogenesis, transcription factor Barx2 [47]. Moreover, single gene knock-outs of *Gdf5 *and *Gdf6 *resulted in defects in multiple elements of the appendicular and craniofacial skeletons, while a more severe skeletal phenotype was observed for the *Gdf5*/*Gdf6 *double knock-out mouse [48]. These findings are consistent with the extremely similar differential expression profiles determined for *Gdf5 *and *Gdf6 *in the present study, in which the expression of both genes peaked significantly at 12.5 dpc (Fig. 5D). By contrast, little is known of the role of Gdf10 in chondrogenesis. Indeed, the only report to date in which *Gdf10 *has been implicated in skeletal biology concerned its expression during bone repair in a model of fracture healing [49]. The steady up-regulation of *Gdf10 *observed between 11.5 dpc and 13.5 dpc in the present study however, suggests that Gdf10 may play an important role in mesenchymal condensation and chondrocyte maturation. Overall, six *Gdf *genes were expressed during *in vivo *chondrogenesis (Fig. 5D). *Gdf6 *was found to be most highly differentially expressed, peaking at 12.5 dpc, although overall expression of *Gdf6 *was very low. Both *Gdf5 *and *Gdf10 *were strongly expressed, and highly differentially expressed. *Gdf1*, although not highly differentially expressed, was found to be expressed strongly throughout *in vivo *chondrogenesis.

It is well established that Wnt proteins mediate important signalling pathways at various stages of chondrogenesis. Accordingly, Wnt gene expression *in vivo *is found to be spatially and temporally restricted within the developing cartilage anlage. *Wnt4 *expression is localized to the developing joints, *Wnt5a *to the perichondrium, and *Wnt5b *to the perichondrium and prehypertrophic chondrocytes [50,51]. Moreover, functional work presented in the same studies demonstrated that Wnt4 promotes chondrocyte hypertrophy, while Wnt5a and Wnt5b operate conversely, by impeding terminal chondrocyte differentiation. Thus, while the data generated by microarray analysis here provides valuable information about relative expression levels, the authors recognise that it cannot provide information regarding the spatial distribution of transcripts within the microdissected tissues. Among the *Wnt *family, only *Wnt11 *and *Wnt5b *were strongly expressed, and only one *Wnt *(*Wnt4*) was found to be highly differentially expressed and dramatically up-regulated between 12.5 dpc and 13.5 dpc (Fig. 5E). Conversely, frizzled-related protein (*Frzb*), a Wnt antagonist, was found to be strongly expressed, and down-regulated between 11.5 dpc and 13.5 dpc (see Additional file 2).

Despite their prominent role in controlling aspects of endochondral bone growth, little is known about the expression or function of Fgf ligands and Fgf receptors within mesenchymal condensations and subsequent cartilage anlagen [52]. Recent work however points to a role for Fgf18 in regulating chondrocyte proliferation and hypertrophy during the earliest stages of chondrogenesis (expression being detected as early as 14.5 dpc) through interaction with Fgfr3 [53]. Our differential expression data (Fig. 5F) support and extend these findings by revealing significant up-regulation of both *Fgf18 *and *Fgfr3 *between 11.5 dpc and 13.5 dpc, and by clarifying the differential expression patterns of *Fgf *ligands and *Fgf *receptors within chondrogenic tissue *in vivo*. Of considerable interest was the finding that *Fgfr4 *expression is significantly up-regulated between 11.5 dpc and 13.5 dpc. Fgfr4 has not previously been associated with chondrogenesis, although it is known to mediate early events in the signalling cascade regulating skeletal muscle development in the developing limb [54]. The *Fgfr4 *differential expression data presented here imply that in addition to the role of Fgf4 in myogenesis, it may also have a role in chondrogenesis.

#### Transcription factors

The first families of transcription factors considered here are the homeo box (*Hox*) genes (Fig. 5G). A large group of genes comprised of four gene clusters, the *Hox *genes were mostly found to be down-regulated from 11.5 dpc to 13.5 dpc, although the opposite trend was observed for some *Hox *genes. Only three members (*Hoxa7*, *Hoxa10*, and *Hoxa11*) of the *Hoxa *gene cluster were strongly expressed. The most highly differentially expressed of these was *Hoxa10*. *Hoxa7 *and *Hoxa11 *were more strongly expressed than *Hoxa10 *however, although neither was significantly differentially expressed. Four *Hoxb *genes were weakly expressed during *in vivo *chondrogenesis, and none of them were highly differentially expressed. Only one *Hoxc *gene (*Hoxc10*) was highly differentially expressed during *in vivo *chondrogenesis, down-regulated by almost 3-fold from 11.5 dpc to 13.5 dpc. None of the other *Hoxc *genes were significantly differentially expressed, although the expression level of *Hoxc9 *was found to be very high throughout the time course. Most of the *Hoxd *gene cluster were very weakly expressed. The most highly down-regulated *Hoxd *genes were generally the more 3' members of the cluster (*Hoxd9*, *Hoxd10*, *Hoxd11*, and *Hoxd13*). While *Hox *genes have established roles in pattern formation, they may play additional roles in chondrogenesis. The overall down-regulation of the more highly expressed members of the *Hox *family suggests that *Hox *gene silencing may be required for the progression of cartilage formation. Transgenic mouse data showing that the persistent expression of *Hoxa2 *in cells entering chondrogenesis impairs cartilage development and causes chondrodysplasia [55], supports this inhibitory role for at least a subset of *Hox *genes. A further early role for Hox genes in regulating the expression of the key chondrogenic transcription factor, *Sox9*, has been shown in recent experiments [56].

Interestingly, while many *Hox *genes were found to be down-regulated in chondrogenic tissue from 11.5 dpc to 13.5 dpc in the present study, three *Hox *genes (*Hoxa7*, *Hoxb5*, and *Hoxc5*) were up-regulated during this time by close to or greater than two-fold. This is the first time that vertebrate limb expression data has been published for *Hoxa7*, *Hoxb5*, and *Hoxc5*.

Eight T-box (*Tbx*) genes were expressed during *in vivo *chondrogenesis (Fig. 5H). Of these *Tbx15*, *Tbx3 *and *Tbx18*, were all strongly expressed and highly down-regulated from 11.5 dpc to 13.5 dpc.

Expression of 16 *Sry*-box (*Sox*) genes was detected during *in vivo *chondrogenesis (Fig. 5I). Of these, four were found to be highly expressed although none of these four genes were significantly differentially expressed. Among these genes were *Sox9 *and *Sox6*. Moderate expression levels were detected for five *Sox *genes and of these, only *Sox8 *was significantly differentially expressed, up-regulated by almost 4-fold between 11.5 dpc and 13.5 dpc. Seven *Sox *genes were found to be expressed weakly during *in vivo *chondrogenesis. Expression data generated in the present study are consistent with the importance of *Sox9 *throughout chondrogenesis [57,58]. *Sox9 *was not highly differentially expressed from 11.5 dpc to 13.5 dpc but its average Log_2 _intensity was found to be extremely high, indicating ubiquitously high expression of *Sox9 *throughout the transition from pre-chondrogenic mesenchymal cell to fully differentiated chondrocyte. It is known that Sox9 exerts its influence over chondrogenesis, in part, by regulating the expression of multiple prominent components of the cartilage matrix including the quintessential chondrocyte marker, *Col2a1 *[12]. This transcriptional activity was found to be largely dependent upon the combined activity of a long form of Sox5 (L-Sox5), and Sox6 [59]. In the present study, *Sox6 *expression closely resembles that of *Sox9 *in that although it was not found to be highly differentially expressed throughout chondrogenesis, its absolute expression level (average Log_2 _intensity) was found to be very high.

Interestingly *Sox5 *expression was quite low in the present study (Fig. 5I). It is possible that this result does not accurately reflect the abundance of *Sox5 *transcripts because of technical limitations with array design. The single probe for *Sox5 *on the microarray is located 1445 bp upstream of the 3'-end of the mouse *Sox5 *coding sequence, and since the amplified RNA used to interrogate to array has an inherent 3' bias, it is likely that this results in an inability to hybridize efficiently.

Our results also demonstrate that additional *Sox *genes, *Sox4, Sox8*, *Sox10, Sox11 and Sox13 *participate in chondrogenesis *in vivo*. Previous studies have shown *Sox4 *expression localized to diaphyseal hypertrophic chondrocytes during the commencement of long bone ossification, and later in the hypertrophic chondrocytes of the embryonic growth plate [60], as well as along the posterior margin, and proximally on the anterior margin of chicken limb buds at a stage equivalent to mouse hindlimb 11.5 dpc [61]. Expression of *Sox8 *and *Sox10 *has also been noted previously in limb development, occurring, like that of *Sox5*, *Sox6*, and *Sox9*, in a manner which essentially prefigures the developing skeleton [62]. Moreover, in the same study it was noted that each of these genes was up-regulated in ectopic cartilages in response to Bmp7, implying roles for Sox8 and Sox10 in chondrogenesis. The highly similar expression patterns detected for *Sox8 *and *Sox10 *here suggest that these genes may have overlapping functions *in vivo*. That *Sox8 *and *Sox10 *are most highly expressed at 13.5 dpc points towards roles for these genes *in vivo *in chondrocyte maturation, or in preparing chondrocytes for subsequent hypertrophy. *Sox13 *was up-regulated from 11.5 dpc to 12.5 dpc, to a level at which it was maintained until 13.5 dpc. The association of Sox13 with limb bud development has recently been demonstrated by immunohistochemistry [63].

Another large transcription factor family of which multiple members were found to exhibit dynamic patterns of differential expression was the forkhead box (*Fox*) gene family (Fig. 5J). Three *Fox *genes were very highly expressed. Notably, *Foxc2 *was significantly up-regulated, almost 3-fold between 11.5 dpc and 12.5 dpc, and approximately 3.5 fold by 13.5 dpc. Furthermore, *Foxo1 *and *Foxp1 *were both strongly expressed, and differentially expressed during *in vivo *chondrogenesis, although the expression patterns of these transcription factors differed markedly. Whereas *Foxo1 *expression was found to increase in a linear fashion by almost 5-fold between 11.5 dpc and 13.5 dpc, *Foxp1 *expression peaked at 12.5 dpc, before dropping by greater than 3-fold by 13.5 dpc. A further four *Fox *genes were moderately expressed. One of these genes, *Foxa3*, was dramatically up-regulated between 11.5 dpc and 13.5 dpc, by over 20-fold. Seven other *Fox *genes were weakly expressed and of these, only *Foxd1 *was differentially expressed, up-regulated more than 4-fold from 11.5 dpc to 13.5 dpc.

Only two *Fox *genes however, have been implicated in skeletogenesis to date. *Foxa3 *transcripts have been detected by *in situ *hybridization in the vertebrae and ribs of 14.5 dpc mouse embryos [64], while expression of *Foxc2 *has been observed during axial and appendicular skeletogenesis in mouse. Interestingly, in the developing limb, it was found that *Foxc2 *transcripts localized to the periphery of the mesenchymal condensation at 12.5 dpc, and the perichondrium of the cartilage anlagen at 13.5 dpc [65]. The function of *Foxa3 *and *Foxc2 *during chondrogenesis remains unknown.

#### Extracellular matrix molecules

Chondrogenesis involves the secretion of a complex extracellular matrix rich in collagens and non-collagenous proteoglycans. Thus, it was expected that many genes encoding components of cartilage extracellular matrix would be dramatically up-regulated during *in vivo *chondrogenesis. The results which follow include the differential expression patterns observed for prominent non-collagenous extracellular matrix proteins as well as for the collagen gene family and the integrin family, which encode extracellular matrix receptors.

As predicted, many genes encoding cartilage extracellular matrix molecules were highly up-regulated (Fig. 5K). It was found that cartilage link protein (*Hapln1*, hyaluronan and proteoglycan link protein 1) was highly expressed, and by far the most highly up-regulated gene during *in vivo *chondrogenesis, its expression having increased approximately 200-fold between 11.5 dpc and 13.5 dpc. Cartilage oligomeric matrix protein (*Comp*), aggrecan (*Agc1*), matrilin 1 (*Matn1*), and chondroitin sulphate proteoglycan 4 (*Cspg4*) were also highly expressed and very highly up-regulated between 11.5 dpc and 13.5 dpc. Matrilin 3 (*Matn3*) and matrilin 2 (*Matn2*) were also very highly up-regulated, although they were expressed only moderately. The most highly expressed of these genes were *Cspg4 *and *Matn4*, although *Matn4 *was not found to be differentially expressed. Fibronectin 1 (*Fn1*) was not differentially expressed, and its expression was moderate. Versican (*Vcan*) expression was down-regulated by over 10-fold from 11.5 dpc to 13.5 dpc while Tenascin C (*TnC*) expression was moderate but was up-regulated by almost 8-fold. As with other components of the cartilage ECM, many collagen genes and integrins were differentially expressed during *in vivo *chondrogenesis (see Additional file 4).

### Ontology approaches identify a large cohort of novel genes expressed during chondrogenesis for further investigation

The importance of this study is realised by the utility of the microarray data as an invaluable resource for investigating the roles of novel genes during *in vivo *chondrogenesis. In order to identify such genes, ontological approaches were undertaken to classify differentially expressed genes according to biological process and thus identify those for which an association with ontologies relating to cartilage and skeletal development has yet to be made. Accordingly, the 931 genes for which a differential expression of greater than three and a average Log_2 _intensity of greater than or equal to six was observed between 11.5 dpc – 13.5 dpc (see Additional file 2) were interrogated by GOstat software using the MGI GO gene association database  in order to identify highly represented gene ontologies. As predicted, ontologies relating to cartilage and skeletal development were observed. Among the down-regulated genes, 9 were classified under 'skeletal development' (GO:0001501). Among the up-regulated genes, 30 were classified under 'skeletal development' (GO:0001501), 20 under 'ossification' (GO:0001503), 15 under 'cartilage development' (GO:0051216), 5 under 'chondrocyte differentiation' (GO:0002062), and 4 under 'cartilage condensation' (GO:0001502). Of these genes, 44 appeared under multiple classifications. Therefore, a total of 39 genes were classified by GOstat analysis as having previously been implicated in cartilage or skeletal development, leaving 892 novel genes for which an ontological association with chondrogenesis has yet to made. Thus, this study has served to identify a large cohort of novel genes whose expression or function during *in vivo *chondrogenesis is uncharacterized and potentially represents important developmental regulators. This dataset is therefore an important reference for future gene discovery and stands as an invaluable resource for ongoing research into skeletal development and disease.

Our studies are the first comprehensive attempt to identify genes which regulate the initiation of chondrogenesis *in vivo*. We have described the differential expression patterns observed for major gene families encoding adhesion molecules, transcription factors, and signalling molecules known to be important in many developmental processes, and have implicated members of these developmental gene families in chondrogenesis for the first time. In addition, we have identified many new candidate genes that may be involved in the initiation of chondrogenesis, on the basis of their differential expression patterns observed here, in combination with their known association with specific biological processes or known molecular function.

## Conclusion

By conducting whole genome microarray analyses of RNA derived specifically from tissues representative of key stages of *in vivo *limb bud chondrogenesis, we have generated and thoroughly validated expression profiles which for the first time define the suite of genes expressed by chondrogenic tissues in the formation of cartilage from pre-condensed mesenchyme during embryogenesis. In doing so, we have defined the expression profiles of known chondrogenically and developmentally important gene families expressed by chondrogenic tissues during cartilage formation, and have identified 931 genes significantly differentially expressed during this process, consisting of large gene cohorts particular to each stage of cartilage development – pre-condensed mesenchyme, mesenchymal condensations, and cartilage anlagen. It is anticipated that these data will be important in further unravelling the fundamental control networks which drive cartilage development, in investigating the molecular pathology of inherited skeletal diseases, and in exploring which properties of pre-skeletal limb bud mesenchymal cells predispose them to efficient chondrogenic differentiation, the understanding of which promises to aid research into therapeutic cartilage repair. Thus, the data-set generated in this study stands as an important development for skeletal biology research.

## Methods

### Microdissection of chondrogenic condensations and preparation of RNA

**Figure 6:**
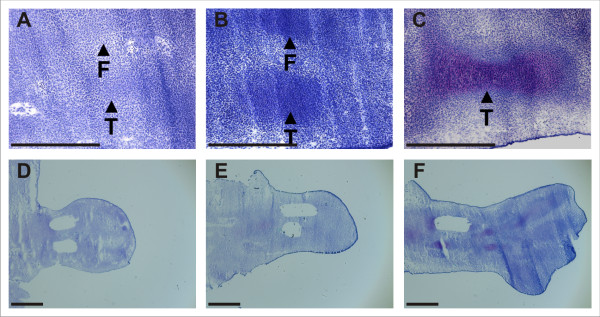
**Mouse hindlimb microdissections**. 11.5 dpc, 12.5 dpc, and 13.5 dpc mouse hindlimb cryosections stained with Toluidine Blue. Upper panels **(A-C) **show intact tissue; lower panels **(D-F) **show cryosections following microdissection of tibial and fibular tissues. Staining of tissues shown in lower panels was performed following microdissection. T = Tibia, F = Fibula. Scale bar = 500 μm.

We have previously reported global differential expression profiles corresponding to zones of the growth plate generated by microarray analysis of RNA derived from tissue microdissected from long bones of Swiss white mice [66]. So that these datasets could be compared with those of the present study, Swiss white mice were used here also. Accordingly, pregnant female Swiss white mice were sacrificed in accordance with Institutional Animal Ethics guidelines and embryos were harvested at 11.5 dpc, 12.5 dpc and 13.5 dpc and transferred to PBS at 4°C. The hind limb buds were promptly dissected and embedded in Tissue-Tek OCT (Sakura Fine Technical), snap-frozen in isopentane and stored at -80°C. 6 μm sections of limb tissue corresponding to the regions of tibial and fibular development were prepared using a cryostat (Leica CM1850), mounted on RNAse-free SuperFrost Plus slides (Biolab Scientific), fixed in 70% ethanol, washed in RNAse-free water, and dehydrated in 70%, 95%, and 100% ethanol for thirty seconds each, and air-dried. Regions corresponding to the mesenchymal condensations of developing tibiae and fibulae were microdissected from slides immobilized on the stage of an inverted light microscope (Leica DM IL) using an ophthalmic scalpel (Feather) fixed to the scanning xy-object guide. Mesenchymal condensations and cartilage anlagen at 12.5 dpc and 13.5 dpc were readily visible in untreated (ie fixed and dehydrated, but not stained) sections by light microscopy, allowing precise microdissection of these tissues. In 12.5 dpc hind limb sections, a prominent blood vessel was found to run between, and perpendicular to the developing tibia and fibula, thus appearing in the transverse plane as the tibia and fibula came into view (Fig. 6). The appearance of this blood vessel was used as a guide for locating the precondensed mesenchymal cells in 11.5 dpc hind limb sections. 231 sections from a total of four 11.5 dpc mouse hind limbs, three 12.5 dpc mouse hind limbs, and four 13.5 dpc mouse hind limbs were microdissected (Fig. 6). The tissue from each time point was pooled into RNase-free Eppendorf tubes and total RNA was extracted using the RNeasy Micro Kit (Qiagen). To assess RNA yield, purity, and integrity, total RNA samples were interrogated by capillary electrophoresis with a Bioanalyzer 2100 (Agilent Technologies), using a Series II RNA 6000 Pico Kit, according to the manufacturer's specifications (Agilent Technologies). All total RNA samples were confirmed to be of high quality, with a RIN of >7.7 (Agilent). Total RNA from tibial and fibular tissue microdissected from each time point was linearly amplified [67] in two rounds using the MessageAmp aRNA kit (Ambion) following the manufacturer's instructions. 100–150 ng of first-round amplified cRNA was used as template for each of the second round amplifications. Quality and yield was assessed by capillary electrophoresis. Amplified RNA samples (1.25 μg) were fluorescently labelled with Cy3 or Cy5 fluorophores (Amersham) according to the manufacturer's instructions, analysed by 1.5% agarose gel electrophoresis, and visualized using a Typhoon fluorescence scanner (Amersham) (see Additional file 5). These data demonstrated that the majority of transcripts in each sample were approximately 150 nt – 400 nt in length, and the lack of low molecular weight fluorescent signal confirmed that all unincorporated dyes were successfully removed during the purification procedure.

### Microarray hybridizations

Interrogation of the 11.5 dpc, 12.5 dpc, and 13.5 dpc cRNA samples by microarray analysis involved a saturated hybridization strategy with dye swaps. Thus, cRNA samples from each time point were labelled with both Cy3 and Cy5 fluorophores, and six hybridizations were performed such that each time point was compared with each other time point using both dye combinations (to normalize against dye biases). 44 K whole mouse genome microarrays (G4122F, ID014868, Agilent) were hybridized according to the manufacturer's instructions. The arrays were then scanned at 10 μm resolution on an Axon 4000B scanner and the features acquired with the GenePix Pro 4.1 software (Axon Instruments). The raw data was then processed using a print-tip loess normalization [68] using limmaGUI [69,70] which is an implemented package of R used to fit linear models to microarray data . Genes were ranked according to their differential expression and gene lists were generated based on biological process and function (GOStat and OntoExpress).

### PCR analysis

Semi-quantitative PCR was used for initial validation of the differential expression of specific genes during hind limb chondrogenesis. Reverse transcription reactions were performed using equal quantities of cRNA as template, to generate cDNA. Equal quantities of cDNA were used as template for PCR amplification of specific genes, using primers designed as close as possible to the end of the 3' untranslated region of the cDNA sequence. To ensure that the reactions were stopped during the linear phase of amplification, reactions were set up in triplicate and routinely removed from the thermocycler at 25 cycles, 30 cycles, and 35 cycles. Quantitative PCR (qPCR) was used for quantitative validation of the differential expression of specific genes during hind limb chondrogenesis. Primers for qPCR were designed using online software . qPCR was performed using the FastStart TaqMan Probe Master real time PCR kit in 10 μl reactions comprised of 5 μl Roche FastStart Probe Master (Rox), 50 ng cDNA, 240 nM additional ROX Reference Dye, 100 nM UPL Probe, and 200 nM each primer. Thermal cycling was conducted on an Applied Biosystems 7700 Real-Time PCR System, as follows: initial denaturation at 95°C for 10 minutes, followed by 50 cycles of denaturation at 95°C for 30 seconds, annealing and polymerization at 60°C for one minute. qPCR data were analyzed using Applied Biosystems SDS 2.2.2 software. All primer sequences are available on request.

### Histology

Toluidine Blue staining with Fast Green counter-staining [71] was used to detect accumulation of proteoglycans in sagittal cryosections of embryonic mouse limb buds, as a histological assay for chondrogenesis.

### *In situ *hybridization

Hindlimbs from E11.5, 12.5 and 13.5 embryos were fixed overnight in 4% PFA then cyroprotected by immersion in 30% sucrose overnight. 12 μm frozen sections were then subjected to *in situ *hybridization for each gene [72].

## Abbreviations

aRNA: amplified RNA; cRNA: complementary RNA; dpc: days post coitum; GO: gene ontology; qPCR: quantitative PCR; RT-PCR: reverse transcription PCR.

## Authors' contributions

TLC carried out all tissue preparations including dissection and microdissection, RNA purification and amplification, semi-quantitative and quantitative PCR analyses, microarray hybridizations (in conjunction with DB), and histology. DB conducted the initial microarray data analysis. PGF designed and generated probes for, and carried out the *in situ *hybridizations. JFB co-ordinated the study. TLC and JFB drafted the manuscript. All authors contributed to the design of the study, and critically reviewed and approved the final manuscript.

## Supplementary Material

Additional file 1 **Major categories of molecular function of genes differentially expressed during chondrogenesis.** The data provided represent the 931 genes significantly (>3-fold) up-regulated **(A) **or down-regulated **(B) **during chondrogenesis (see Additional file 2 for gene list) after mining using OntoExpress gene ontology software and clustering according biological process. Click here for file

Additional file 2 **List of genes differentially up- or down-regulated (>3-fold) during chondrogenesis.** The data provided represent the cohort of genes whose Log_2 _intensity (A) was above background level, and whose expression changed by greater than 3-fold during chondrogenesis in the mouse limb between 11.5 dpc and 13.5 dpc. Click here for file

Additional file 3 **Validation of microarray expression data by comparative literature analysis.** The data provided represent the differential expression profiles for a list of 50 genes whose differential expression during chondrogenesis have been corroborated elsewhere in the scientific literature, and includes specific references to which the reader is directed for each gene. Click here for file

Additional file 4 **Differential expression of collagens and integrins during chondrogenesis.** The data provided represent the differential expression profiles of the **(A) **collagens, **B) **alpha-integrins (*Itga*), and **C) **beta-integrins (*Itgb*) during chondrogenesis in the mouse limb between 11.5 dpc and 13.5 dpc. Yellow bars indicate relative expression at 11.5 dpc. Blue bars indicate relative expression at 12.5 dpc. Orange bars indicate relative expression at 13.5 dpc. For each gene, the time point at which the relative expression was lowest was defined as one, and this was used to calculate the relative expression at the remaining time points. Numbers above each gene denote average Log_2 _intensity (A) for that gene. Click here for file

Additional file 5 **Agarose gel electrophoresis for cRNA samples from 11.5 dpc, 12.5 dpc, or 13.5 dpc following labelling with Cy3 or Cy5 fluorophores.** This agarose gel indicates successful labelling of the cRNA samples from 11.5 dpc – 13.5 dpc mouse limb chondrogenic tissues with Cy3 or Cy5 fluorophores, and verifies that unincorporated dyes were removed from the cRNA samples during purification. Click here for file
