# Supplementary material for: Global comparative transcriptome analysis of cartilage formation in vivo
Source: BMC Dev Biol. 2009 Mar 10;9:20. doi: 10.1186/1471-213X-9-20 (PMC2662817; doi:10.1186/1471-213X-9-20)
Supplement: Additional file 2 — List of genes differentially up- or down-regulated (>3-fold) during chondrogenesis. The data provided represent the cohort of genes whose Log2 intensity (A) was above background level, and whose expression changed by greater than 3-fold during chondrogenesis in the mouse limb between 11.5 dpc and 13.5 dpc. [file 1471-213X-9-20-S2.doc]

| **NCBI Accession** | **Gene Name** | **Microarray Probe ID** | **11.5dpc** | **12.5dpc** | **13.5dpc** | **A** |
| --- | --- | --- | --- | --- | --- | --- |
| **Down-regulated genes:** | |  |  |  |  |  |
| BC039744 | Acp1 | A_52_P589735 | 3.5 | 2.7 | 1.0 | 8.9 |
| NM_013758 | Add3 | A_52_P167249 | 3.6 | 1.8 | 1.0 | 8.3 |
| BC049786 | Alx4 | A_52_P522640 | 4.4 | 2.2 | 1.0 | 7.6 |
| AK129277 | Amot | A_51_P321385 | 6.0 | 2.5 | 1.0 | 11.8 |
| NM_024213 | Anapc4 | A_52_P237451 | 3.0 | 1.6 | 1.0 | 6.8 |
| AK144017 | Ankrd52 | A_52_P647069 | 3.1 | 1.1 | 1.0 | 6.8 |
| NM_023210 | Anp32e | A_51_P400581 | 3.2 | 1.9 | 1.0 | 6.2 |
| NM_176837 | Arhgap18 | A_52_P671769 | 3.0 | 1.5 | 1.0 | 6.7 |
| NM_027871 | Arhgef3 | A_51_P380337 | 7.0 | 2.5 | 1.0 | 7.8 |
| BC082312 | Arid5b | A_52_P432580 | 6.5 | 2.6 | 1.0 | 10.3 |
| NM_007491 | Art5 | A_51_P160787 | 5.5 | 2.9 | 1.0 | 7.8 |
| NM_033474 | Arvcf | A_52_P583031 | 4.2 | 2.5 | 1.0 | 8.5 |
| NM_023048 | Asb4 | A_51_P332998 | 4.7 | 1.2 | 1.0 | 6.3 |
| NM_007495 | Astn1 | A_51_P143951 | 11.2 | 5.8 | 1.0 | 8.0 |
| BC049640 | Atp5f1 | A_52_P106929 | 3.5 | 1.8 | 1.0 | 7.8 |
| AK009185 | Barx2 | A_52_P533817 | 3.8 | 2.0 | 1.0 | 9.1 |
| NM_016707 | Bcl11a | A_51_P263004 | 4.8 | 2.9 | 1.0 | 11.5 |
| NM_027208 | Bdh2 | A_51_P470935 | 3.4 | 2.8 | 1.0 | 8.5 |
| NM_212457 | Bex4 | A_51_P494342 | 3.5 | 2.6 | 1.0 | 12.5 |
| NM_138313 | Bmf | A_52_P384394 | 6.5 | 2.6 | 1.0 | 10.8 |
| NM_007552 | Bmi1 | A_52_P39157 | 3.0 | 2.1 | 1.0 | 9.3 |
| NM_007560 | Bmpr1b | A_52_P586944 | 6.1 | 3.6 | 1.0 | 7.7 |
| NM_172618 | Btbd9 | A_52_P542860 | 14.7 | 13.2 | 1.0 | 8.9 |
| NM_024285 | Bves | A_52_P337126 | 5.2 | 2.4 | 1.0 | 7.9 |
| NM_027332 | Bxdc5 | A_52_P517006 | 4.0 | 1.9 | 1.0 | 8.3 |
| NM_145621 | Camkv | A_52_P497208 | 6.3 | 3.7 | 1.0 | 6.6 |
| XM_355539 | Camta1 | A_52_P642005 | 5.7 | 3.1 | 1.0 | 6.6 |
| NM_011797 | Car14 | A_51_P175424 | 8.2 | 5.2 | 1.0 | 10.0 |
| NM_009801 | Car2 | A_51_P455647 | 4.6 | 1.6 | 1.0 | 7.7 |
| NM_145398 | Casd1 | A_51_P496462 | 3.1 | 1.8 | 1.0 | 11.4 |
| NM_009810 | Casp3 | A_51_P382618 | 3.6 | 2.5 | 1.0 | 9.2 |
| NM_023052 | Ccl21c | A_51_P129480 | 8.8 | 2.4 | 1.0 | 7.1 |
| NM_007635 | Ccng2 | A_51_P360165 | 4.4 | 3.0 | 1.0 | 11.2 |
| NM_172839 | Ccnj | A_51_P418016 | 3.5 | 2.1 | 1.0 | 8.2 |
| NM_007639 | Cd1d1 | A_51_P517430 | 6.9 | 3.2 | 1.0 | 9.5 |
| NM_009846 | Cd24a | A_52_P244193 | 3.0 | 1.7 | 1.0 | 13.2 |
| NM_007667 | Cdh8 | A_51_P290886 | 6.3 | 9.2 | 1.0 | 6.2 |
| AK037032 | Cecr2 | A_52_P260468 | 4.5 | 2.8 | 1.0 | 6.1 |
| NM_019405 | Cetn2 | A_51_P516297 | 3.9 | 2.4 | 1.0 | 7.3 |
| NM_010828 | Cited2 | A_51_P105837 | 4.5 | 2.5 | 1.0 | 11.5 |
| NM_019563 | Cited4 | A_51_P109099 | 3.3 | 1.4 | 1.0 | 6.3 |
| NM_025809 | Clec14a | A_52_P566665 | 3.1 | 2.0 | 1.0 | 8.2 |
| NM_053155 | Clmn | A_51_P200667 | 3.1 | 2.1 | 1.0 | 9.1 |
| NM_009931 | Col4a1 | A_51_P124254 | 3.1 | 2.2 | 1.0 | 10.9 |
| NM_009939 | Cops2 | A_52_P259087 | 3.2 | 4.1 | 1.0 | 6.0 |
| NM_001024698 | Cpa2 | A_52_P320261 | 3.1 | 2.7 | 1.0 | 8.6 |
| NM_007759 | Crabp2 | A_51_P521283 | 5.1 | 2.3 | 1.0 | 6.4 |
| NM_172728 | Creb5 | A_52_P267564 | 16.1 | 10.9 | 1.0 | 10.2 |
| NM_007765 | Crmp1 | A_52_P42255 | 4.3 | 2.5 | 1.0 | 8.7 |
| NM_016669 | Crym | A_51_P264695 | 4.3 | 4.1 | 1.0 | 7.9 |
| NM_146087 | Csnk1a1 | A_51_P336491 | 3.1 | 2.6 | 1.0 | 11.8 |
| D16263 | Cspg2 | A_52_P251703 | 9.6 | 6.0 | 1.0 | 10.5 |
| NM_007791 | Csrp1 | A_52_P209184 | 5.1 | 1.0 | 1.4 | 8.5 |
| NM_212450 | Ctdspl2 | A_52_P581138 | 3.0 | 3.5 | 1.0 | 6.6 |
| NM_009982 | Ctsc | A_52_P195839 | 4.4 | 2.5 | 1.0 | 8.8 |
| AK164640 | Cul4b | A_52_P213181 | 3.1 | 2.2 | 1.0 | 10.8 |
| NM_013655 | Cxcl12 | A_51_P172502 | 4.6 | 3.4 | 1.0 | 11.9 |
| NM_019932 | Cxcl4 | A_51_P441426 | 3.6 | 3.5 | 1.0 | 8.8 |
| NM_023785 | Cxcl7 | A_51_P428372 | 7.2 | 6.3 | 1.0 | 6.3 |
| NM_133769 | Cyfip2 | A_52_P536947 | 3.2 | 1.2 | 1.0 | 7.8 |
| NM_025967 | D16Ertd472e | A_51_P412732 | 3.1 | 1.2 | 1.0 | 6.0 |
| BC035277 | D1Ertd471e | A_51_P452533 | 4.7 | 2.4 | 1.0 | 6.8 |
| NM_177259 | Dab1 | A_52_P392216 | 7.5 | 3.1 | 1.0 | 7.3 |
| BC050903 | Dcamkl1 | A_52_P26991 | 4.2 | 2.1 | 1.0 | 9.2 |
| NM_027539 | Dcamkl2 | A_51_P165834 | 3.7 | 1.9 | 1.0 | 8.8 |
| NM_007832 | Dck | A_51_P500718 | 3.8 | 2.6 | 1.0 | 7.1 |
| NM_010025 | Dcx | A_52_P244349 | 3.6 | 2.3 | 1.0 | 7.8 |
| NM_016765 | Ddah2 | A_51_P341177 | 3.4 | 2.4 | 1.0 | 13.3 |
| NM_001004364 | Ddef2 | A_52_P536731 | 4.1 | 1.6 | 1.0 | 7.1 |
| NM_030096 | Ddx52 | A_51_P136479 | 5.8 | 2.9 | 1.0 | 6.7 |
| NM_007839 | Dhx15 | A_51_P247960 | 3.1 | 2.1 | 1.0 | 12.0 |
| AK147699 | Dlg5 | A_52_P482888 | 3.7 | 2.3 | 1.0 | 6.9 |
| NM_007868 | Dmd | A_52_P81210 | 4.2 | 1.6 | 1.0 | 6.1 |
| NM_008298 | Dnaja1 | A_52_P310225 | 3.1 | 2.1 | 1.0 | 8.6 |
| AB159607 | Dnm3os | A_52_P590898 | 3.5 | 2.9 | 1.0 | 11.3 |
| NM_007872 | Dnmt3a | A_52_P169048 | 3.9 | 2.4 | 1.0 | 9.2 |
| NM_170778 | Dpyd | A_51_P473259 | 9.0 | 2.0 | 1.0 | 6.1 |
| AK012530 | Dusp4 | A_51_P347333 | 3.0 | 1.3 | 1.0 | 7.7 |
| NM_026268 | Dusp6 | A_51_P502614 | 3.3 | 2.8 | 1.0 | 12.4 |
| NM_025943 | Dzip1 | A_52_P196458 | 4.6 | 3.2 | 1.0 | 9.3 |
| NM_007897 | Ebf1 | A_51_P188054 | 3.7 | 2.2 | 1.0 | 9.3 |
| NM_010096 | Ebf3 | A_51_P246543 | 5.1 | 4.4 | 1.0 | 11.8 |
| NM_010336 | Edg2 | A_51_P475573 | 3.0 | 2.1 | 1.0 | 11.7 |
| NM_010101 | Edg3 | A_52_P502577 | 3.3 | 1.7 | 1.0 | 10.1 |
| NM_010111 | Efnb2 | A_52_P340073 | 3.5 | 1.8 | 1.0 | 7.5 |
| NM_028133 | Egln3 | A_52_P387009 | 4.2 | 1.6 | 1.0 | 6.8 |
| NM_145973 | Ell3 | A_52_P382926 | 6.5 | 3.4 | 1.0 | 7.8 |
| BC005527 | Enpp3 | A_51_P332917 | 6.0 | 3.2 | 1.0 | 6.0 |
| NM_010141 | Epha7 | A_52_P504787 | 6.3 | 3.0 | 1.0 | 9.2 |
| NM_011808 | Ets1 | A_52_P194250 | 4.1 | 1.6 | 1.0 | 8.8 |
| AK033517 | Evi1 | A_52_P418901 | 35.2 | 6.8 | 1.0 | 7.7 |
| NM_007971 | Ezh2 | A_51_P248067 | 3.8 | 2.6 | 1.0 | 10.1 |
| NM_183221 | Fat4 | A_52_P675530 | 3.3 | 2.7 | 1.0 | 11.7 |
| NM_133754 | Fblim1 | A_51_P309370 | 6.5 | 3.2 | 1.0 | 8.0 |
| NM_172591 | Fcho2 | A_52_P50455 | 3.1 | 1.9 | 1.0 | 6.4 |
| NM_139232 | Fgd4 | A_52_P639229 | 3.8 | 2.9 | 1.0 | 6.9 |
| NM_010212 | Fhl2 | A_51_P140237 | 4.3 | 2.6 | 1.0 | 8.9 |
| NM_021891 | Fignl1 | A_52_P148553 | 3.3 | 2.3 | 1.0 | 10.4 |
| NM_008026 | Fli1 | A_52_P821 | 3.2 | 3.7 | 1.0 | 11.2 |
| BC029674 | Flt1 | A_52_P307922 | 3.5 | 1.8 | 1.0 | 6.3 |
| AK080318 | Fmnl2 | A_51_P222936 | 5.2 | 2.6 | 1.0 | 11.8 |
| NM_153118 | Fnbp1l | A_52_P113000 | 3.4 | 2.7 | 1.0 | 12.4 |
| NM_207636 | Fndc3a | A_52_P33800 | 3.5 | 1.8 | 1.0 | 7.0 |
| NM_013522 | Frg1 | A_51_P267544 | 3.0 | 2.4 | 1.0 | 8.9 |
| NM_172475 | Frmd4a | A_52_P477559 | 3.2 | 2.5 | 1.0 | 11.8 |
| NM_011356 | Frzb | A_51_P286748 | 4.7 | 1.6 | 1.0 | 11.1 |
| BC043060 | Fusip1 | A_52_P478532 | 3.2 | 2.0 | 1.0 | 10.3 |
| NM_207669 | Gabpb1 | A_52_P331701 | 3.6 | 2.2 | 1.0 | 6.9 |
| NM_008086 | Gas1 | A_51_P157083 | 19.8 | 12.0 | 1.0 | 10.2 |
| NM_008087 | Gas2 | A_51_P355629 | 8.0 | 6.8 | 1.0 | 7.6 |
| NM_008376 | Gimap1 | A_51_P507509 | 3.9 | 1.4 | 1.0 | 6.3 |
| NM_008121 | Gja5 | A_52_P525317 | 5.5 | 3.5 | 1.0 | 8.4 |
| NM_010298 | Glrb | A_51_P250797 | 8.3 | 3.2 | 1.0 | 7.5 |
| NM_198620 | Gm440 | A_52_P619192 | 3.7 | 2.4 | 1.0 | 7.6 |
| NM_001007580 | Gm784 | A_52_P89884 | 3.5 | 2.2 | 1.0 | 11.3 |
| XM_129042 | Gm96 | A_51_P220262 | 5.4 | 4.5 | 1.0 | 8.2 |
| NM_175668 | Gpr4 | A_52_P128691 | 3.2 | 1.6 | 1.0 | 6.7 |
| NM_010351 | Gsc | A_51_P370717 | 6.6 | 5.4 | 1.0 | 9.5 |
| NM_010357 | Gsta4 | A_51_P112223 | 3.4 | 3.0 | 1.0 | 7.9 |
| AK002213 | Gstm7 | A_51_P114002 | 3.2 | 1.5 | 1.0 | 8.0 |
| U83174 | Gt(ROSA)26Sor | A_51_P426919 | 3.2 | 2.2 | 1.0 | 8.2 |
| NM_001039519 | Gtf2a2 | A_51_P320377 | 3.6 | 1.9 | 1.0 | 8.1 |
| BC028539 | H2afv | A_51_P472630 | 3.1 | 3.0 | 1.0 | 14.1 |
| NM_008210 | H3f3a | A_52_P1041253 | 5.3 | 2.4 | 1.0 | 11.3 |
| NM_172473 | Hace1 | A_51_P512947 | 4.5 | 1.9 | 1.0 | 7.7 |
| NM_010405 | Hba-x | A_51_P204053 | 8.4 | 2.5 | 1.0 | 12.0 |
| NM_008219 | Hbb-bh1 | A_51_P179672 | 21.9 | 5.9 | 1.0 | 11.3 |
| NM_008221 | Hbb-y | A_51_P350058 | 9.4 | 3.3 | 1.0 | 12.6 |
| NM_008224 | Hcfc1 | A_52_P424138 | 7.3 | 3.3 | 1.0 | 6.7 |
| NM_008229 | Hdac2 | A_51_P116007 | 3.5 | 1.7 | 1.0 | 6.1 |
| NM_013886 | Hdgfrp3 | A_51_P410581 | 3.8 | 2.7 | 1.0 | 8.5 |
| NM_008234 | Hells | A_51_P351970 | 3.5 | 2.4 | 1.0 | 9.0 |
| NM_010417 | Heph | A_51_P466270 | 8.8 | 4.2 | 1.0 | 8.2 |
| NM_028705 | Herc3 | A_51_P249313 | 3.9 | 3.1 | 1.0 | 8.4 |
| NM_010431 | Hif1a | A_51_P387608 | 3.2 | 1.6 | 1.0 | 9.5 |
| NM_030082 | Hist3h2ba | A_51_P338664 | 4.1 | 4.6 | 1.0 | 10.7 |
| BC016539 | Hmcn1 | A_51_P393518 | 4.5 | 2.1 | 1.0 | 7.9 |
| NM_010439 | Hmgb1 | A_52_P323044 | 3.5 | 1.9 | 1.0 | 6.5 |
| BC090989 | Hmgb1-rs17 | A_52_P602787 | 4.0 | 3.2 | 1.0 | 11.4 |
| NM_008253 | Hmgb3 | A_51_P138548 | 3.9 | 2.3 | 1.0 | 13.5 |
| NM_016884 | Hnrpc | A_52_P218537 | 5.4 | 2.4 | 1.0 | 9.2 |
| NM_016690 | Hnrpdl | A_52_P292693 | 3.2 | 2.3 | 1.0 | 8.0 |
| NM_021510 | Hnrph1 | A_51_P299195 | 3.4 | 2.0 | 1.0 | 13.3 |
| NM_144802 | Hnrpll | A_51_P390676 | 3.5 | 2.4 | 1.0 | 8.7 |
| AK051789 | Hook1 | A_52_P212597 | 4.4 | 3.1 | 1.0 | 8.3 |
| NM_008263 | Hoxa10 | A_51_P466285 | 5.1 | 3.1 | 1.0 | 9.9 |
| NM_008273 | Hoxd11 | A_51_P112319 | 7.2 | 2.3 | 1.0 | 6.0 |
| NM_010469 | Hoxd4 | A_51_P260265 | 5.6 | 1.8 | 1.0 | 7.7 |
| XM_355338 | Hoxd8 | A_52_P485542 | 3.5 | 2.0 | 1.0 | 6.4 |
| NM_013555 | Hoxd9 | A_52_P385584 | 5.5 | 2.4 | 1.0 | 7.8 |
| NM_010474 | Hs3st1 | A_51_P365152 | 3.6 | 2.9 | 1.0 | 6.0 |
| NM_008300 | Hspa4 | A_52_P617817 | 3.1 | 2.2 | 1.0 | 6.1 |
| NM_030244 | Ier5l | A_52_P562641 | 4.0 | 3.0 | 1.0 | 9.9 |
| NM_009879 | Ift81 | A_52_P459276 | 3.4 | 1.9 | 1.0 | 6.5 |
| NM_010512 | Igf1 | A_51_P147942 | 10.5 | 4.1 | 1.0 | 8.8 |
| NM_008342 | Igfbp2 | A_51_P423308 | 4.2 | 2.9 | 1.0 | 10.1 |
| NM_033608 | Igsf9 | A_52_P443701 | 3.5 | 1.9 | 1.0 | 6.2 |
| NM_019440 | Iigp2 | A_51_P416295 | 3.5 | 3.2 | 1.0 | 6.0 |
| NM_178112 | Ints8 | A_52_P103977 | 4.3 | 2.2 | 1.0 | 7.2 |
| NM_021459 | Isl1 | A_52_P337246 | 6.0 | 2.4 | 1.0 | 9.6 |
| XM_129010 | Jakmip2 | A_52_P304056 | 3.1 | 2.2 | 1.0 | 7.2 |
| NM_010591 | Jun | A_51_P325914 | 11.6 | 8.1 | 1.0 | 10.4 |
| NM_021452 | Kcnmb4 | A_51_P452714 | 3.3 | 1.8 | 1.0 | 10.9 |
| BC087623 | Kctd1 | A_51_P437847 | 3.8 | 2.1 | 1.0 | 9.5 |
| NM_021099 | Kit | A_51_P355906 | 5.0 | 2.3 | 1.0 | 6.9 |
| AK054351 | Klhl24 | A_51_P362698 | 4.2 | 2.5 | 1.0 | 7.0 |
| NM_172787 | L3mbtl3 | A_51_P115940 | 5.5 | 2.6 | 1.0 | 7.3 |
| NM_021295 | Lancl1 | A_52_P270899 | 3.6 | 1.4 | 1.0 | 6.7 |
| XM_129479 | Lct | A_52_P164797 | 3.4 | 2.1 | 1.0 | 6.3 |
| NM_010195 | Lgr5 | A_51_P218091 | 4.0 | 3.3 | 1.0 | 7.6 |
| NM_010714 | Lhx9 | A_51_P330481 | 5.1 | 2.7 | 1.0 | 10.7 |
| NM_001031772 | Lin28b | A_52_P676861 | 9.4 | 2.8 | 1.0 | 6.6 |
| NM_001039354 | Lin7a | A_52_P48762 | 3.1 | 4.4 | 1.0 | 6.4 |
| XM_888040 | Lphn2 | A_52_P605480 | 3.3 | 1.7 | 1.0 | 8.4 |
| NM_030695 | Lrba | A_51_P338837 | 7.5 | 2.7 | 1.0 | 9.0 |
| NM_008377 | Lrig1 | A_51_P116137 | 4.2 | 2.1 | 1.0 | 9.4 |
| NM_028915 | Lrrcc1 | A_52_P174840 | 4.3 | 2.4 | 1.0 | 6.2 |
| NM_028815 | Lrriq2 | A_52_P357181 | 5.3 | 6.3 | 1.0 | 6.4 |
| NM_008516 | Lrrn1 | A_51_P412966 | 3.5 | 3.9 | 1.0 | 8.9 |
| NM_025968 | Ltb4dh | A_51_P444437 | 5.1 | 2.8 | 1.0 | 11.8 |
| NM_145100 | Lypd1 | A_51_P497395 | 5.0 | 1.6 | 1.0 | 7.2 |
| NM_010750 | Mab21l1 | A_52_P620448 | 14.6 | 3.5 | 1.0 | 8.0 |
| NM_011839 | Mab21l2 | A_51_P327206 | 7.6 | 3.5 | 1.0 | 8.0 |
| NM_010367 | Magi1 | A_51_P406346 | 3.4 | 2.4 | 1.0 | 9.4 |
| NM_030153 | Mak10 | A_52_P501969 | 3.3 | 2.1 | 1.0 | 6.8 |
| NM_008538 | Marcks | A_51_P352968 | 3.2 | 2.3 | 1.0 | 12.9 |
| NM_010807 | Marcksl1 | A_51_P257550 | 5.4 | 2.7 | 1.0 | 13.6 |
| NM_148922 | Mdm1 | A_52_P327236 | 3.9 | 2.6 | 1.0 | 9.7 |
| NM_019946 | Mgst1 | A_52_P549827 | 10.0 | 4.2 | 1.0 | 8.7 |
| AK011386 | Mllt3 | A_52_P1066847 | 3.5 | 3.3 | 1.0 | 7.4 |
| NM_026178 | Mmd | A_51_P431470 | 4.9 | 2.2 | 1.0 | 8.9 |
| NM_008606 | Mmp11 | A_51_P293087 | 3.9 | 2.6 | 1.0 | 11.3 |
| NM_007863 | Mpp3 | A_51_P497039 | 3.3 | 2.1 | 1.0 | 6.2 |
| NM_008209 | Mr1 | A_51_P160913 | 3.1 | 1.8 | 1.0 | 6.2 |
| NM_010825 | Mrg1 | A_51_P422429 | 13.5 | 7.7 | 1.0 | 9.8 |
| NM_013601 | Msx2 | A_51_P388661 | 3.2 | 1.3 | 1.0 | 7.7 |
| NM_016804 | Mtx2 | A_51_P391847 | 3.6 | 2.0 | 1.0 | 8.6 |
| NM_033597 | Myb | A_51_P375543 | 3.1 | 3.4 | 1.0 | 8.0 |
| NM_008709 | Mycn | A_52_P536494 | 4.1 | 3.2 | 1.0 | 11.3 |
| NM_175260 | Myh10 | A_52_P680681 | 3.4 | 2.0 | 1.0 | 12.3 |
| NM_030557 | Mynn | A_52_P242227 | 3.9 | 1.6 | 1.0 | 6.9 |
| NM_010863 | Myo1b | A_52_P289325 | 3.5 | 2.2 | 1.0 | 6.8 |
| Y18276 | Nbea | A_52_P236671 | 4.8 | 1.4 | 1.0 | 6.8 |
| NM_175329 | Ndg2 | A_51_P269084 | 3.2 | 2.2 | 1.0 | 7.3 |
| NM_010890 | Nedd4 | A_51_P295022 | 5.6 | 2.9 | 1.0 | 10.5 |
| NM_008685 | Nfe2 | A_51_P515965 | 3.9 | 1.5 | 1.0 | 6.7 |
| AK034793 | Nfib | A_52_P282494 | 3.8 | 2.3 | 1.0 | 6.7 |
| NM_138314 | Nme7 | A_52_P464629 | 3.7 | 2.1 | 1.0 | 7.2 |
| NM_010923 | Nnat | A_52_P484956 | 7.6 | 4.3 | 1.0 | 11.2 |
| NM_010929 | Notch4 | A_51_P195875 | 3.4 | 2.2 | 1.0 | 6.7 |
| NM_009697 | Nr2f2 | A_52_P620399 | 3.5 | 2.4 | 1.0 | 9.8 |
| NM_026746 | Nsmce2 | A_51_P389386 | 3.6 | 3.0 | 1.0 | 8.1 |
| BC033459 | Numb | A_52_P517905 | 3.8 | 2.1 | 1.0 | 6.4 |
| AK147579 | Odz4 | A_52_P144368 | 3.8 | 2.6 | 1.0 | 10.1 |
| NM_010098 | Opn3 | A_52_P215176 | 4.0 | 2.5 | 1.0 | 6.4 |
| NM_011959 | Orc5l | A_51_P214825 | 4.7 | 2.2 | 1.0 | 6.1 |
| NM_027881 | Osbpl3 | A_51_P196844 | 3.2 | 1.8 | 1.0 | 8.7 |
| NM_011859 | Osr1 | A_52_P63728 | 8.7 | 4.9 | 1.0 | 8.0 |
| NM_020595 | Otor | A_51_P205524 | 4.8 | 4.9 | 1.0 | 8.8 |
| XM_204283 | Palld | A_52_P33192 | 3.1 | 3.4 | 1.0 | 6.2 |
| NM_146240 | Pamci | A_52_P596592 | 4.7 | 1.9 | 1.0 | 7.1 |
| AK053576 | Pard3 | A_51_P189198 | 3.1 | 2.1 | 1.0 | 6.5 |
| NM_011041 | Pax9 | A_52_P593379 | 3.1 | 1.9 | 1.0 | 6.1 |
| AK144446 | Pbx1 | A_52_P111385 | 4.0 | 2.2 | 1.0 | 7.4 |
| AK122503 | Pcdh10 | A_51_P209150 | 9.1 | 4.6 | 1.0 | 8.4 |
| AK147265 | Pcdh17 | A_51_P131747 | 3.7 | 2.4 | 1.0 | 7.4 |
| NM_018764 | Pcdh7 | A_51_P260740 | 3.3 | 2.0 | 1.0 | 6.8 |
| NM_021543 | Pcdh8 | A_51_P274488 | 18.6 | 8.9 | 1.0 | 10.0 |
| AY861425 | Pcdh9 | A_52_P197199 | 4.3 | 2.6 | 1.0 | 10.9 |
| NM_008791 | Pcp4 | A_51_P253984 | 3.5 | 8.0 | 1.0 | 11.6 |
| NM_008802 | Pde7a | A_51_P293269 | 3.5 | 2.1 | 1.0 | 10.6 |
| NM_011058 | Pdgfra | A_51_P345649 | 3.4 | 2.5 | 1.0 | 12.4 |
| AK079187 | Peli2 | A_51_P350666 | 3.9 | 1.7 | 1.0 | 8.0 |
| NM_011070 | Pfdn2 | A_51_P327778 | 3.6 | 2.6 | 1.0 | 10.5 |
| NM_028273 | Pgam5 | A_51_P194022 | 3.1 | 1.5 | 1.0 | 7.6 |
| NM_198419 | Phactr1 | A_51_P182572 | 3.1 | 1.8 | 1.0 | 7.6 |
| NM_027642 | Phf6 | A_52_P209992 | 3.0 | 2.3 | 1.0 | 10.8 |
| NM_153412 | Phldb2 | A_52_P153700 | 6.2 | 4.0 | 1.0 | 9.6 |
| XM_129968 | Phlpp | A_51_P156158 | 3.4 | 1.8 | 1.0 | 6.1 |
| NM_181585 | Pik3r3 | A_52_P607103 | 5.5 | 3.1 | 1.0 | 10.0 |
| NM_011097 | Pitx1 | A_52_P522255 | 3.5 | 2.3 | 1.0 | 14.0 |
| NM_008862 | Pkia | A_52_P314129 | 5.8 | 2.4 | 1.0 | 7.9 |
| NM_001029838 | Pknox2 | A_52_P6442 | 4.8 | 3.2 | 1.0 | 8.9 |
| NM_175175 | Plekhf2 | A_51_P138790 | 3.0 | 1.5 | 1.0 | 7.9 |
| NM_152804 | Plk2 | A_51_P290576 | 6.4 | 5.6 | 1.0 | 10.8 |
| NM_173169 | Plk4 | A_52_P635976 | 5.2 | 2.6 | 1.0 | 6.5 |
| NM_012040 | Pnck | A_51_P270324 | 3.2 | 1.9 | 1.0 | 6.4 |
| NM_001033225 | Pnrc1 | A_52_P619820 | 3.2 | 2.8 | 1.0 | 12.7 |
| XM_979374 | Ppil5 | A_51_P347240 | 3.2 | 2.7 | 1.0 | 9.6 |
| NM_011563 | Prdx2 | A_51_P147207 | 3.1 | 2.1 | 1.0 | 14.0 |
| NM_028410 | Prkrir | A_52_P380649 | 3.2 | 1.4 | 1.0 | 6.7 |
| AK016009 | Prr3 | A_52_P391825 | 3.7 | 2.3 | 1.0 | 6.7 |
| NM_011127 | Prrx1 | A_52_P483713 | 3.1 | 2.1 | 1.0 | 10.0 |
| NM_028707 | Psd2 | A_51_P157577 | 3.0 | 1.9 | 1.0 | 6.6 |
| NM_011968 | Psma6 | A_51_P428364 | 3.6 | 2.4 | 1.0 | 10.5 |
| NM_019550 | Ptbp2 | A_51_P363818 | 3.4 | 1.7 | 1.0 | 9.8 |
| NM_008973 | Ptn | A_52_P10793 | 3.8 | 2.6 | 1.0 | 11.6 |
| NM_011203 | Ptpn12 | A_51_P447668 | 3.5 | 1.7 | 1.0 | 6.5 |
| AK012957 | Punc | A_51_P436559 | 6.1 | 2.4 | 1.0 | 7.6 |
| NM_026405 | Rab32 | A_51_P293688 | 11.4 | 6.6 | 1.0 | 10.7 |
| NM_019491 | Rala | A_52_P672740 | 4.2 | 1.9 | 1.0 | 7.5 |
| NM_177721 | Ranbp6 | A_52_P450123 | 3.1 | 1.6 | 1.0 | 6.6 |
| NM_019930 | Ranbp9 | A_51_P106191 | 3.2 | 1.6 | 1.0 | 8.1 |
| XM_130987 | Rarres1 | A_51_P401184 | 5.2 | 1.9 | 1.0 | 8.5 |
| NM_181318 | Rasgef1b | A_52_P188827 | 6.0 | 1.5 | 1.0 | 10.3 |
| AK004534 | Rasl11b | A_51_P293938 | 3.3 | 2.1 | 1.0 | 12.4 |
| NM_134083 | Rcbtb2 | A_52_P444637 | 3.8 | 2.4 | 1.0 | 8.0 |
| NM_009041 | Rdx | A_52_P571684 | 3.1 | 1.4 | 1.0 | 6.2 |
| NM_009051 | Rex2 | A_52_P412109 | 4.0 | 2.8 | 1.0 | 7.6 |
| NM_009061 | Rgs2 | A_51_P419768 | 3.7 | 3.3 | 1.0 | 9.5 |
| XM_620516 | Rkhd3 | A_52_P102248 | 8.6 | 5.1 | 1.0 | 12.4 |
| NM_028810 | Rnd3 | A_52_P343839 | 3.1 | 2.3 | 1.0 | 10.7 |
| NM_175136 | Rnf122 | A_52_P636742 | 4.2 | 2.6 | 1.0 | 10.2 |
| AK151379 | Rnf180 | A_51_P215530 | 3.5 | 2.0 | 1.0 | 6.9 |
| NM_019413 | Robo1 | A_51_P174314 | 3.3 | 2.0 | 1.0 | 9.4 |
| NM_133669 | Rp2h | A_52_P558751 | 3.0 | 2.2 | 1.0 | 6.7 |
| NM_023396 | Rprm | A_51_P278653 | 7.2 | 3.1 | 1.0 | 8.2 |
| NM_172815 | Rspo2 | A_51_P222337 | 14.7 | 1.7 | 1.0 | 6.6 |
| NM_153457 | Rtn1 | A_52_P424959 | 3.2 | 1.5 | 1.0 | 6.7 |
| NM_009822 | Runx1t1 | A_52_P612137 | 3.5 | 3.6 | 1.0 | 11.9 |
| NM_178280 | Sall3 | A_52_P405655 | 11.3 | 2.0 | 1.0 | 6.1 |
| NM_021788 | Sap30 | A_51_P432199 | 3.2 | 1.9 | 1.0 | 9.6 |
| NM_145587 | Sbk1 | A_51_P498388 | 3.1 | 1.8 | 1.0 | 9.7 |
| NM_138741 | Sdpr | A_51_P277336 | 3.1 | 2.1 | 1.0 | 7.9 |
| NM_025483 | Senp7 | A_52_P88722 | 3.3 | 1.6 | 1.0 | 6.2 |
| NM_009144 | Sfrp2 | A_51_P520849 | 16.6 | 9.9 | 1.0 | 11.4 |
| NM_146083 | Sfrs7 | A_51_P466673 | 3.8 | 1.9 | 1.0 | 8.2 |
| NM_019989 | Sh3bgrl | A_52_P426513 | 3.0 | 1.9 | 1.0 | 10.3 |
| NM_009189 | Six1 | A_52_P586679 | 7.2 | 5.3 | 1.0 | 9.9 |
| NM_172371 | Slc16a13 | A_51_P373428 | 4.6 | 2.3 | 1.0 | 6.6 |
| NM_172653 | Slc39a10 | A_51_P223498 | 3.4 | 1.6 | 1.0 | 6.3 |
| NM_011403 | Slc4a1 | A_51_P196972 | 3.6 | 1.8 | 1.0 | 8.5 |
| NM_175499 | Slitrk6 | A_51_P503822 | 4.5 | 1.0 | 1.0 | 6.9 |
| NM_008017 | Smc2 | A_51_P265660 | 3.8 | 1.7 | 1.0 | 6.2 |
| BC007174 | Smc5 | A_51_P489909 | 6.2 | 2.9 | 1.0 | 7.2 |
| NM_028887 | Smchd1 | A_52_P322017 | 3.1 | 2.0 | 1.0 | 7.0 |
| NM_022316 | Smoc1 | A_51_P443403 | 4.3 | 3.1 | 1.0 | 6.9 |
| NM_009227 | Snrpe | A_51_P363634 | 3.0 | 2.2 | 1.0 | 13.3 |
| NM_026998 | Snx6 | A_52_P85875 | 3.7 | 1.4 | 1.0 | 6.8 |
| AK004158 | Sox11 | A_52_P160955 | 3.2 | 2.4 | 1.0 | 13.2 |
| NM_011441 | Sox17 | A_52_P279368 | 5.7 | 2.4 | 1.0 | 6.5 |
| NM_011446 | Sox7 | A_51_P476018 | 3.3 | 2.6 | 1.0 | 6.9 |
| NM_009239 | Sp4 | A_52_P481477 | 3.5 | 2.2 | 1.0 | 7.2 |
| NM_011464 | Spint2 | A_51_P497350 | 3.8 | 5.3 | 1.0 | 8.8 |
| NM_023689 | Spock3 | A_51_P237153 | 20.0 | 8.2 | 1.0 | 6.2 |
| NM_028358 | Ssbp1 | A_52_P55053 | 3.3 | 1.7 | 1.0 | 9.7 |
| NM_024186 | Ssbp2 | A_51_P408932 | 3.3 | 2.8 | 1.0 | 11.9 |
| NM_023672 | Ssbp3 | A_51_P109668 | 3.5 | 2.3 | 1.0 | 13.1 |
| NM_009183 | St8sia4 | A_51_P236267 | 4.0 | 1.2 | 1.0 | 8.6 |
| NM_019641 | Stmn1 | A_51_P495641 | 3.1 | 2.4 | 1.0 | 14.4 |
| AK031650 | Stxbp4 | A_52_P401783 | 3.0 | 1.6 | 1.0 | 6.4 |
| NM_172294 | Sulf1 | A_51_P142744 | 6.9 | 10.2 | 1.0 | 11.1 |
| NM_022724 | Suv39h2 | A_52_P16931 | 3.0 | 1.8 | 1.0 | 7.1 |
| BC082586 | Syne2 | A_51_P224872 | 5.0 | 2.8 | 1.0 | 10.3 |
| NM_028052 | Synpr | A_51_P376656 | 5.1 | 3.4 | 1.0 | 7.1 |
| NM_018804 | Syt11 | A_51_P260730 | 3.2 | 2.5 | 1.0 | 11.4 |
| NM_172804 | Syt16 | A_52_P149521 | 4.3 | 1.8 | 1.0 | 6.4 |
| BC094568 | Taf1 | A_51_P139400 | 3.3 | 2.1 | 1.0 | 7.1 |
| NM_025816 | Tax1bp1 | A_52_P71496 | 4.2 | 1.7 | 1.0 | 6.9 |
| NM_029564 | Tax1bp3 | A_51_P487384 | 3.3 | 1.6 | 1.0 | 11.7 |
| NM_025706 | Tbc1d15 | A_52_P120904 | 3.0 | 1.5 | 1.0 | 6.0 |
| NM_009323 | Tbx15 | A_52_P232802 | 3.6 | 2.6 | 1.0 | 12.3 |
| NM_023814 | Tbx18 | A_51_P438711 | 8.9 | 4.9 | 1.0 | 10.5 |
| NM_011535 | Tbx3 | A_52_P183368 | 6.6 | 1.9 | 1.0 | 10.8 |
| AK028407 | Tbx4 | A_52_P16899 | 5.1 | 3.9 | 1.0 | 9.2 |
| NM_011537 | Tbx5 | A_52_P523712 | 6.7 | 4.2 | 1.0 | 6.8 |
| NM_001002241 | Tdrd1 | A_52_P48218 | 3.0 | 1.5 | 1.0 | 6.9 |
| NM_011569 | Tekt1 | A_51_P328890 | 3.3 | 2.4 | 1.0 | 6.9 |
| NM_011570 | Tes | A_52_P74441 | 5.9 | 2.9 | 1.0 | 6.5 |
| NM_011585 | Tia1 | A_52_P21822 | 3.2 | 2.0 | 1.0 | 7.7 |
| NM_009386 | Tjp1 | A_51_P390739 | 3.9 | 1.7 | 1.0 | 7.1 |
| AY155196 | Tle1 | A_52_P330694 | 3.0 | 2.9 | 1.0 | 9.9 |
| NM_177794 | Tmem26 | A_52_P126403 | 10.4 | 2.3 | 1.0 | 7.8 |
| NM_178715 | Tmem30b | A_52_P547612 | 4.7 | 3.1 | 1.0 | 8.7 |
| NM_025284 | Tmsb10 | A_51_P419711 | 6.7 | 2.2 | 1.0 | 8.6 |
| XM_001000101 | Tnks2 | A_51_P434527 | 3.2 | 3.5 | 1.0 | 6.1 |
| NM_172913 | Tnrc9 | A_51_P465082 | 8.7 | 4.6 | 1.0 | 8.8 |
| NM_011623 | Top2a | A_51_P252157 | 3.7 | 3.9 | 1.0 | 8.9 |
| NM_145711 | Tox | A_51_P421912 | 3.3 | 2.1 | 1.0 | 10.2 |
| XM_888341 | Trio | A_52_P521507 | 5.0 | 2.8 | 1.0 | 12.1 |
| NM_133975 | Trip12 | A_52_P407918 | 3.6 | 2.3 | 1.0 | 9.1 |
| NM_173378 | Trp53bp2 | A_51_P363564 | 3.0 | 2.1 | 1.0 | 8.6 |
| NM_021897 | Trp53inp1 | A_51_P175580 | 4.6 | 2.8 | 1.0 | 10.7 |
| NM_019510 | Trpc3 | A_51_P317695 | 3.3 | 1.9 | 1.0 | 7.0 |
| BC058264 | Tshz1 | A_51_P394833 | 4.9 | 2.1 | 1.0 | 11.7 |
| NM_173007 | Tspan12 | A_51_P374707 | 4.2 | 2.9 | 1.0 | 11.0 |
| NM_197996 | Tspan15 | A_52_P657376 | 3.0 | 2.4 | 1.0 | 6.5 |
| NM_009442 | Ttf1 | A_52_P205522 | 3.1 | 2.2 | 1.0 | 6.4 |
| NM_015767 | Ttpa | A_52_P29610 | 3.1 | 1.8 | 1.0 | 6.5 |
| NM_009458 | Ube2b | A_51_P483557 | 3.2 | 1.7 | 1.0 | 8.5 |
| NM_018798 | Ubqln2 | A_52_P381929 | 3.0 | 1.9 | 1.0 | 9.4 |
| AK122422 | Unc13b | A_51_P177242 | 3.5 | 1.5 | 1.0 | 7.2 |
| NM_026573 | Upf3b | A_52_P595162 | 3.1 | 2.0 | 1.0 | 8.3 |
| NM_144879 | Vash2 | A_51_P452203 | 3.1 | 2.1 | 1.0 | 6.8 |
| NM_177683 | Vgll4 | A_52_P205797 | 3.1 | 1.8 | 1.0 | 7.8 |
| NM_016982 | Vpreb1 | A_51_P400217 | 4.5 | 4.3 | 1.0 | 8.0 |
| AK084751 | Vps13a | A_51_P130874 | 3.2 | 2.2 | 1.0 | 7.1 |
| NM_027260 | Vrk2 | A_52_P200350 | 3.3 | 1.6 | 1.0 | 6.2 |
| NM_016757 | Wbp1 | A_51_P160372 | 3.1 | 2.4 | 1.0 | 12.5 |
| NM_028866 | Wdr33 | A_52_P59612 | 3.2 | 1.9 | 1.0 | 6.3 |
| NM_001039388 | Wdr37 | A_52_P434052 | 3.6 | 1.7 | 1.0 | 7.5 |
| NM_026323 | Wfdc2 | A_51_P465211 | 4.2 | 3.1 | 1.0 | 11.7 |
| NM_019653 | Wsb1 | A_51_P271370 | 3.2 | 1.8 | 1.0 | 6.2 |
| L04961 | Xist | A_51_P145453 | 3.9 | 3.1 | 1.0 | 11.4 |
| NM_013771 | Yme1l1 | A_52_P444849 | 3.6 | 2.2 | 1.0 | 6.9 |
| NM_023249 | Ypel1 | A_51_P265016 | 3.1 | 2.3 | 1.0 | 9.6 |
| NM_146090 | Zadh2 | A_51_P137388 | 3.7 | 1.9 | 1.0 | 11.2 |
| NM_175472 | Zcchc11 | A_52_P382040 | 3.6 | 3.5 | 1.0 | 7.3 |
| NM_025893 | Zcchc18 | A_51_P350748 | 8.1 | 5.0 | 1.0 | 9.1 |
| NM_022985 | Zfand6 | A_51_P304859 | 3.1 | 1.5 | 1.0 | 7.7 |
| NM_021559 | Zfp191 | A_52_P664559 | 3.3 | 2.6 | 1.0 | 6.3 |
| NM_023685 | Zfp306 | A_51_P278214 | 3.1 | 1.7 | 1.0 | 7.1 |
| AK077686 | Zfp367 | A_52_P250543 | 3.0 | 1.7 | 1.0 | 7.1 |
| NM_029952 | Zfp422-rs1 | A_52_P239023 | 3.5 | 2.2 | 1.0 | 6.2 |
| NM_033327 | Zfp423 | A_51_P328439 | 3.5 | 2.2 | 1.0 | 8.9 |
| NM_177811 | Zfp459 | A_51_P452576 | 3.7 | 2.6 | 1.0 | 6.0 |
| AK173266 | Zfp462 | A_52_P655285 | 3.1 | 2.3 | 1.0 | 10.3 |
| NM_207255 | Zfp532 | A_52_P342131 | 3.3 | 1.6 | 1.0 | 8.2 |
| NM_026856 | Zfp644 | A_51_P447454 | 3.1 | 2.2 | 1.0 | 8.3 |
| NM_001024928 | Zfp667 | A_52_P532957 | 3.3 | 1.6 | 1.0 | 7.3 |
| **Up-regulated genes:** |  |  |  |  |  |  |
| NM_212441 | Acsm3 | A_51_P487175 | 1.0 | 1.5 | 4.9 | 7.1 |
| NM_009606 | Acta1 | A_51_P246854 | 1.0 | 1.2 | 3.3 | 6.0 |
| AK028812 | Adamts12 | A_52_P150851 | 1.0 | 1.5 | 6.1 | 8.7 |
| NM_175643 | Adamts2 | A_51_P227222 | 1.0 | 2.1 | 10.0 | 6.4 |
| CK790536 | Adamts3 | A_51_P462407 | 1.0 | 2.0 | 16.7 | 9.5 |
| NM_009624 | Adcy9 | A_51_P501538 | 1.0 | 1.4 | 8.2 | 10.0 |
| NM_007416 | Adra1b | A_52_P639048 | 1.0 | 6.1 | 6.4 | 7.8 |
| NM_007424 | Agc1 | A_51_P462271 | 1.0 | 4.8 | 30.0 | 11.9 |
| NM_018862 | Agpat1 | A_52_P200465 | 1.0 | 1.6 | 3.6 | 9.0 |
| NM_001039959 | Ahnak | A_52_P496566 | 1.0 | 1.8 | 3.0 | 8.1 |
| XM_884305 | Ak5 | A_51_P274259 | 1.0 | 1.6 | 3.6 | 6.8 |
| NM_007431 | Akp2 | A_51_P494125 | 1.0 | 4.1 | 34.9 | 7.9 |
| NM_009657 | Aldoc | A_51_P220681 | 1.0 | 1.4 | 3.9 | 10.5 |
| NM_019496 | Ammecr1 | A_52_P305995 | 1.0 | 1.8 | 4.2 | 9.7 |
| NM_007426 | Angpt2 | A_51_P201982 | 1.0 | 1.0 | 5.1 | 6.4 |
| NM_010730 | Anxa1 | A_51_P283590 | 1.0 | 7.2 | 35.8 | 9.0 |
| NM_013469 | Anxa11 | A_51_P280597 | 1.0 | 1.1 | 7.7 | 7.0 |
| NM_013471 | Anxa4 | A_51_P256342 | 1.0 | 1.1 | 3.3 | 9.3 |
| NM_009673 | Anxa5 | A_51_P426754 | 1.0 | 1.6 | 3.5 | 13.0 |
| NM_013473 | Anxa8 | A_51_P207591 | 1.0 | 1.3 | 3.2 | 10.5 |
| NM_007461 | Apba2 | A_51_P173858 | 1.0 | 1.8 | 13.2 | 7.7 |
| AK157407 | Apcdd1 | A_51_P175018 | 1.0 | 1.1 | 4.2 | 9.5 |
| NM_007472 | Aqp1 | A_51_P125205 | 1.0 | 1.4 | 6.5 | 7.3 |
| NM_007486 | Arhgdib | A_52_P515247 | 1.0 | 1.9 | 4.5 | 8.4 |
| NM_023142 | Arpc1b | A_51_P331279 | 1.0 | 2.0 | 3.9 | 13.5 |
| BC020108 | Arsi | A_51_P384136 | 1.0 | 2.4 | 17.5 | 8.9 |
| NM_029291 | Ascc2 | A_51_P121228 | 1.0 | 1.8 | 3.3 | 10.2 |
| NM_025711 | Aspn | A_51_P142813 | 1.0 | 4.7 | 13.4 | 6.5 |
| NM_026482 | Atp2b1 | A_52_P348986 | 1.0 | 1.0 | 3.6 | 11.0 |
| NM_027106 | Avpi1 | A_51_P261087 | 1.0 | 1.5 | 3.4 | 11.6 |
| NM_029792 | B3gat1 | A_52_P17982 | 1.0 | 1.9 | 3.7 | 6.8 |
| NM_174991 | Bai1 | A_51_P295846 | 1.0 | 1.8 | 3.3 | 6.7 |
| NM_177580 | Baiap2l2 | A_52_P511798 | 1.0 | 1.0 | 6.5 | 8.1 |
| NM_026505 | Bambi | A_51_P248819 | 1.0 | 2.0 | 4.1 | 12.1 |
| U87957 | Bapx1 | A_52_P90603 | 1.0 | 2.9 | 5.6 | 6.1 |
| NM_007529 | Bcan | A_51_P230175 | 1.0 | 2.2 | 3.5 | 6.4 |
| NM_001024468 | Bcat1 | A_52_P507537 | 1.0 | 1.5 | 3.3 | 11.2 |
| NM_009741 | Bcl2 | A_52_P534870 | 1.0 | 2.7 | 3.0 | 12.3 |
| NM_033601 | Bcl3 | A_51_P136542 | 1.0 | 1.5 | 3.1 | 7.1 |
| NM_009744 | Bcl6 | A_52_P161495 | 1.0 | 1.4 | 11.8 | 7.9 |
| NM_175177 | Bdh1 | A_51_P163106 | 1.0 | 1.0 | 3.4 | 10.6 |
| NM_031397 | Bicc1 | A_51_P356493 | 1.0 | 3.2 | 12.9 | 11.5 |
| NM_007555 | Bmp5 | A_51_P516637 | 1.0 | 2.3 | 3.1 | 7.2 |
| NM_007556 | Bmp6 | A_52_P413646 | 1.0 | 1.5 | 9.6 | 6.4 |
| NM_007557 | Bmp7 | A_52_P278354 | 1.0 | 3.8 | 13.2 | 10.5 |
| NM_007558 | Bmp8a | A_51_P213266 | 1.0 | 2.0 | 50.8 | 7.3 |
| NM_145613 | C1qtnf5 | A_51_P373911 | 1.0 | 1.4 | 6.6 | 7.5 |
| NM_027416 | Calml3 | A_51_P137709 | 1.0 | 3.3 | 21.4 | 9.6 |
| NM_009793 | Camk4 | A_52_P672960 | 1.0 | 1.9 | 5.5 | 7.1 |
| NM_007621 | Cbr2 | A_51_P238425 | 1.0 | 2.7 | 3.7 | 9.5 |
| NM_173047 | Cbr3 | A_51_P481159 | 1.0 | 2.6 | 10.8 | 9.2 |
| NM_028763 | Cbx6 | A_51_P391764 | 1.0 | 1.6 | 3.3 | 11.6 |
| NM_021609 | Ccbp2 | A_52_P222073 | 1.0 | 2.8 | 7.1 | 6.5 |
| NM_029779 | Ccdc116 | A_51_P403243 | 1.0 | 1.5 | 3.3 | 6.2 |
| NM_009831 | Ccng1 | A_51_P161612 | 1.0 | 1.3 | 3.9 | 6.9 |
| NM_017466 | Ccrl2 | A_51_P232682 | 1.0 | 1.6 | 4.9 | 6.5 |
| NM_153098 | Cd109 | A_51_P349546 | 1.0 | 1.3 | 4.9 | 7.3 |
| NM_010818 | Cd200 | A_52_P254095 | 1.0 | 3.6 | 21.9 | 8.5 |
| NM_007656 | Cd82 | A_51_P393934 | 1.0 | 1.3 | 3.1 | 9.1 |
| NM_009856 | Cd83 | A_51_P199135 | 1.0 | 5.0 | 4.2 | 10.6 |
| NM_007657 | Cd9 | A_51_P320852 | 1.0 | 5.0 | 9.8 | 12.6 |
| AK030810 | Cdk6 | A_51_P489779 | 1.7 | 1.0 | 5.6 | 11.5 |
| NM_007669 | Cdkn1a | A_51_P363947 | 1.0 | 1.6 | 16.2 | 8.9 |
| NM_009876 | Cdkn1c | A_51_P339540 | 1.0 | 1.0 | 3.5 | 14.0 |
| NM_007670 | Cdkn2b | A_52_P498241 | 1.0 | 1.6 | 8.6 | 7.4 |
| U19596 | Cdkn2c | A_51_P513719 | 1.0 | 2.0 | 3.9 | 8.4 |
| NM_007671 | Cdkn2c | A_51_P513720 | 1.0 | 1.8 | 3.5 | 8.9 |
| NM_009883 | Cebpb | A_52_P63553 | 1.0 | 2.0 | 3.2 | 10.1 |
| NM_207298 | Ceecam1 | A_52_P559545 | 1.0 | 1.4 | 6.8 | 7.8 |
| NM_207223 | Centb5 | A_52_P129114 | 1.0 | 1.4 | 3.3 | 8.9 |
| NM_027180 | Centd2 | A_51_P437247 | 1.0 | 1.6 | 3.9 | 8.0 |
| NM_009888 | Cfh | A_52_P309381 | 1.0 | 3.2 | 23.8 | 6.9 |
| BC023116 | Cgref1 | A_51_P372550 | 1.0 | 1.5 | 15.8 | 9.4 |
| NM_007689 | Chad | A_51_P480073 | 1.0 | 4.2 | 15.0 | 9.0 |
| NM_023850 | Chst1 | A_51_P157840 | 1.0 | 2.1 | 11.4 | 9.6 |
| NM_021439 | Chst11 | A_51_P275101 | 1.0 | 1.4 | 5.3 | 10.8 |
| NM_021528 | Chst12 | A_52_P176409 | 1.0 | 1.3 | 4.1 | 8.5 |
| NM_007702 | Cidea | A_51_P199168 | 1.0 | 4.0 | 11.3 | 7.7 |
| NM_175451 | Ckap4 | A_52_P32121 | 1.0 | 1.3 | 3.7 | 13.2 |
| NM_009131 | Clec11a | A_51_P359800 | 1.0 | 1.7 | 7.2 | 8.2 |
| NM_027085 | Clic3 | A_51_P236755 | 1.0 | 1.4 | 7.2 | 6.2 |
| NM_172469 | Clic6 | A_52_P447284 | 1.0 | 5.0 | 3.4 | 7.8 |
| NM_022319 | Clstn2 | A_52_P424784 | 1.0 | 1.8 | 5.2 | 6.9 |
| NM_026066 | Cmtm5 | A_51_P438924 | 1.0 | 3.6 | 19.0 | 11.1 |
| NM_007729 | Col11a1 | A_51_P459477 | 1.0 | 4.4 | 21.3 | 13.5 |
| NM_009926 | Col11a2 | A_51_P264666 | 1.0 | 4.0 | 33.9 | 11.6 |
| NM_007731 | Col13a1 | A_51_P240374 | 1.0 | 2.3 | 5.3 | 7.1 |
| NM_181277 | Col14a1 | A_51_P326529 | 1.0 | 4.0 | 11.1 | 7.8 |
| NM_009928 | Col15a1 | A_51_P159792 | 1.0 | 1.5 | 7.0 | 7.5 |
| NM_028266 | Col16a1 | A_51_P291062 | 1.0 | 2.6 | 15.7 | 11.1 |
| NM_007742 | Col1a1 | A_52_P525107 | 1.0 | 3.0 | 17.6 | 12.8 |
| NM_007743 | Col1a2 | A_51_P182303 | 1.0 | 1.2 | 3.2 | 14.0 |
| NM_027770 | Col24a1 | A_51_P408430 | 1.0 | 1.8 | 14.8 | 8.3 |
| AK003879 | Col27a1 | A_52_P480088 | 1.0 | 1.1 | 3.2 | 9.8 |
| NM_031163 | Col2a1 | A_51_P317141 | 1.0 | 1.6 | 5.8 | 13.8 |
| XM_897036 | Col6a3 | A_52_P479262 | 1.0 | 2.7 | 15.3 | 12.2 |
| NM_199473 | Col8a2 | A_52_P302544 | 1.0 | 9.0 | 20.3 | 10.9 |
| NM_007740 | Col9a1 | A_51_P231036 | 1.0 | 1.3 | 4.0 | 10.8 |
| NM_007741 | Col9a2 | A_51_P401504 | 1.0 | 1.8 | 6.1 | 13.9 |
| NM_009936 | Col9a3 | A_52_P197963 | 1.0 | 1.5 | 7.0 | 11.3 |
| NM_130449 | Colec12 | A_52_P527625 | 1.0 | 2.0 | 7.1 | 7.9 |
| NM_016685 | Comp | A_51_P409010 | 1.0 | 5.8 | 101.4 | 11.0 |
| BF100712 | Copz2 | A_52_P180310 | 1.0 | 2.0 | 3.2 | 7.6 |
| NM_177834 | Cpa6 | A_51_P390038 | 1.0 | 4.0 | 28.9 | 6.1 |
| NM_025815 | Cpne8 | A_51_P427563 | 1.0 | 2.6 | 19.7 | 7.5 |
| NM_019696 | Cpxm1 | A_51_P139108 | 1.0 | 2.2 | 3.2 | 12.6 |
| NM_024223 | Crip2 | A_51_P314397 | 1.0 | 1.6 | 3.5 | 12.1 |
| NM_019922 | Crtap | A_51_P201721 | 1.0 | 1.5 | 3.7 | 12.7 |
| NM_030004 | Cryl1 | A_51_P336952 | 1.0 | 1.4 | 4.1 | 10.8 |
| NM_139001 | Cspg4 | A_51_P160824 | 1.0 | 3.7 | 15.9 | 12.6 |
| NM_010217 | Ctgf | A_51_P157042 | 1.0 | 13.0 | 29.9 | 13.0 |
| AK003674 | Cthrc1 | A_52_P453785 | 1.0 | 2.6 | 3.5 | 13.0 |
| NM_007802 | Ctsk | A_51_P448236 | 1.0 | 2.9 | 5.2 | 6.5 |
| NM_022325 | Ctsz | A_51_P256202 | 1.0 | 1.5 | 5.6 | 12.2 |
| NM_009142 | Cx3cl1 | A_51_P196925 | 1.0 | 3.3 | 6.1 | 7.9 |
| XM_910566 | Cybrd1 | A_52_P124734 | 1.0 | 1.5 | 4.9 | 8.6 |
| NM_010516 | Cyr61 | A_51_P252859 | 1.0 | 1.5 | 3.2 | 11.4 |
| NM_138686 | Cys1 | A_51_P109258 | 1.0 | 2.1 | 8.3 | 6.1 |
| BC063103 | Cyt1 | A_52_P631547 | 1.0 | 6.1 | 22.3 | 12.2 |
| AK047398 | D10Bwg1379e | A_52_P309084 | 1.0 | 1.2 | 10.8 | 6.4 |
| NM_030250 | D10Ertd438e | A_51_P292030 | 1.0 | 1.4 | 3.3 | 8.7 |
| NM_026790 | D12Ertd647e | A_52_P86693 | 1.0 | 1.9 | 5.2 | 8.2 |
| NM_001001566 | D1Bwg1363e | A_51_P153904 | 1.0 | 1.6 | 4.9 | 10.6 |
| NM_033079 | D6Mm5e | A_51_P503352 | 1.0 | 2.0 | 5.7 | 8.1 |
| NM_007833 | Dcn | A_51_P334104 | 1.0 | 1.3 | 3.3 | 8.2 |
| BC060266 | Ddah1 | A_51_P307168 | 1.0 | 1.5 | 4.9 | 7.9 |
| NM_030143 | Ddit4l | A_52_P311853 | 1.0 | 1.5 | 3.7 | 6.8 |
| AK080874 | Depdc6 | A_52_P661503 | 1.0 | 2.8 | 8.6 | 8.8 |
| NM_007857 | Dhh | A_52_P232637 | 1.0 | 1.2 | 7.3 | 6.4 |
| NM_025522 | Dhrs7 | A_51_P312437 | 1.0 | 1.8 | 5.4 | 9.9 |
| L12721 | Dlk1 | A_51_P135618 | 1.0 | 3.7 | 13.7 | 12.9 |
| NM_010056 | Dlx5 | A_51_P261051 | 1.0 | 3.0 | 5.0 | 10.9 |
| AK035336 | Dock8 | A_51_P484718 | 1.0 | 3.0 | 28.3 | 6.2 |
| NM_010070 | Dok1 | A_51_P275976 | 1.0 | 1.8 | 3.4 | 10.3 |
| NM_009468 | Dpysl3 | A_51_P115441 | 1.0 | 3.3 | 6.5 | 10.4 |
| NM_007878 | Drd4 | A_51_P283499 | 1.0 | 2.6 | 4.6 | 9.9 |
| NM_207649 | Dscr1l1 | A_52_P414464 | 1.2 | 1.0 | 4.4 | 6.9 |
| AK077574 | Dsp | A_51_P101460 | 1.0 | 1.2 | 3.2 | 6.5 |
| NM_007884 | Dspg3 | A_52_P303274 | 1.0 | 3.7 | 39.3 | 8.6 |
| NM_007898 | Ebp | A_51_P429682 | 1.0 | 1.6 | 3.1 | 12.0 |
| NM_010103 | Edil3 | A_52_P325527 | 1.0 | 2.9 | 5.9 | 9.6 |
| NM_010110 | Efnb1 | A_51_P388478 | 1.0 | 1.6 | 3.7 | 11.9 |
| NM_207655 | Egfr | A_52_P106259 | 1.0 | 4.9 | 3.8 | 8.5 |
| NM_007913 | Egr1 | A_51_P367866 | 1.0 | 1.1 | 3.2 | 7.4 |
| NM_020578 | Ehd3 | A_51_P360918 | 1.0 | 1.2 | 3.2 | 11.0 |
| NM_019422 | Elovl1 | A_51_P120305 | 1.0 | 1.3 | 3.1 | 11.7 |
| NM_182840 | Emilin3 | A_52_P45616 | 1.0 | 1.7 | 4.8 | 9.6 |
| NM_010128 | Emp1 | A_52_P120037 | 1.0 | 3.0 | 6.5 | 11.8 |
| NM_010129 | Emp3 | A_51_P446510 | 1.0 | 1.8 | 4.2 | 11.6 |
| NM_028013 | Endod1 | A_51_P505823 | 1.0 | 2.7 | 5.4 | 10.5 |
| NM_008813 | Enpp1 | A_51_P231499 | 1.0 | 2.0 | 4.2 | 9.2 |
| NM_178676 | Entpd3 | A_51_P512992 | 1.0 | 2.1 | 33.8 | 7.7 |
| NM_134065 | Epdr2 | A_52_P577388 | 1.0 | 1.4 | 3.3 | 9.5 |
| NM_010139 | Epha2 | A_52_P518997 | 1.0 | 2.4 | 5.7 | 8.7 |
| NM_010140 | Epha3 | A_52_P276955 | 1.0 | 1.6 | 19.6 | 6.6 |
| NM_007962 | Eva1 | A_52_P322421 | 1.0 | 2.0 | 3.3 | 11.2 |
| NM_019578 | Extl1 | A_51_P346893 | 1.0 | 2.9 | 5.7 | 10.3 |
| NM_010174 | Fabp3 | A_51_P167535 | 1.0 | 2.0 | 16.3 | 9.4 |
| NM_019699 | Fads2 | A_51_P364609 | 1.0 | 1.6 | 3.6 | 9.9 |
| NM_021890 | Fads3 | A_51_P464029 | 1.0 | 1.4 | 4.3 | 10.3 |
| NM_007992 | Fbln2 | A_52_P590535 | 1.0 | 1.4 | 4.5 | 13.7 |
| NM_013517 | Fcer2a | A_51_P210591 | 1.0 | 3.2 | 12.5 | 7.1 |
| NM_008005 | Fgf18 | A_51_P249957 | 1.0 | 1.4 | 3.7 | 8.9 |
| NM_026218 | Fgfr1op2 | A_51_P440838 | 1.0 | 1.4 | 3.9 | 8.8 |
| NM_008011 | Fgfr4 | A_51_P408100 | 1.0 | 1.6 | 4.7 | 10.3 |
| NM_010218 | Fjx1 | A_51_P355589 | 1.0 | 1.1 | 3.6 | 6.7 |
| NM_010221 | Fkbp10 | A_51_P216965 | 1.0 | 1.5 | 3.4 | 12.8 |
| NM_024169 | Fkbp11 | A_52_P326399 | 1.0 | 1.7 | 3.3 | 11.5 |
| NM_012056 | Fkbp9 | A_52_P37681 | 1.0 | 1.8 | 4.7 | 13.1 |
| XM_985863 | Fndc1 | A_51_P153423 | 1.0 | 2.0 | 49.3 | 7.5 |
| NM_145927 | Fntb | A_51_P517680 | 1.0 | 1.7 | 3.1 | 7.5 |
| NM_008260 | Foxa3 | A_52_P64707 | 1.0 | 6.4 | 20.5 | 8.3 |
| NM_008242 | Foxd1 | A_51_P237783 | 1.0 | 3.3 | 5.0 | 7.6 |
| NM_019739 | Foxo1 | A_51_P138044 | 1.0 | 2.6 | 4.9 | 11.0 |
| AK220506 | Fry | A_52_P513041 | 1.0 | 3.4 | 27.0 | 8.4 |
| NM_008557 | Fxyd3 | A_52_P377791 | 1.0 | 1.7 | 3.9 | 8.0 |
| NM_022007 | Fxyd7 | A_51_P379698 | 1.0 | 4.2 | 3.8 | 7.5 |
| AK021164 | Fzd9 | A_51_P511015 | 1.0 | 2.0 | 7.9 | 11.2 |
| XM_194378 | g1l | A_51_P249193 | 1.0 | 2.0 | 4.8 | 6.7 |
| NM_011817 | Gadd45g | A_51_P315904 | 1.0 | 1.0 | 5.5 | 11.7 |
| NM_178389 | Gale | A_51_P401193 | 1.0 | 1.4 | 5.9 | 11.7 |
| NM_134189 | Galnt10 | A_51_P343566 | 1.0 | 1.8 | 3.2 | 10.5 |
| NM_173442 | Gcnt1 | A_52_P21550 | 1.0 | 5.0 | 10.0 | 8.5 |
| NM_020619 | Gcs1 | A_52_P536481 | 1.0 | 1.6 | 4.5 | 13.2 |
| NM_145741 | Gdf10 | A_51_P185247 | 1.0 | 3.0 | 4.0 | 10.8 |
| NM_010280 | Gfra3 | A_51_P520426 | 1.0 | 1.8 | 5.5 | 8.1 |
| NM_011820 | Ggtla1 | A_51_P201709 | 1.0 | 2.0 | 4.7 | 6.3 |
| NM_009149 | Glg1 | A_51_P260548 | 1.0 | 1.6 | 3.6 | 13.6 |
| NM_146211 | Glt25d1 | A_52_P346581 | 1.0 | 1.4 | 3.9 | 11.9 |
| NM_177756 | Glt25d2 | A_51_P331429 | 1.0 | 2.5 | 7.0 | 7.8 |
| NM_001033288 | Gm106 | A_52_P22611 | 1.0 | 1.6 | 5.5 | 7.3 |
| NM_001037750 | Gm1533 | A_52_P435009 | 1.0 | 3.2 | 3.7 | 7.3 |
| XM_111398 | Gm22 | A_52_P560146 | 1.0 | 2.8 | 11.9 | 10.1 |
| BC031445 | Golph3 | A_52_P244162 | 1.0 | 1.9 | 4.2 | 7.3 |
| NM_025872 | Golt1b | A_52_P37665 | 1.0 | 3.0 | 3.4 | 8.7 |
| NM_001001999 | Gp1bb | A_51_P151576 | 1.1 | 1.0 | 4.5 | 6.8 |
| NM_001002268 | Gpr126 | A_52_P414645 | 1.0 | 3.1 | 21.8 | 7.5 |
| NM_001025381 | Gpr17 | A_51_P170463 | 1.0 | 1.1 | 21.0 | 6.4 |
| NM_029771 | Gpr30 | A_51_P185939 | 1.0 | 1.3 | 4.2 | 8.4 |
| NM_175012 | Grp | A_51_P356055 | 1.0 | 3.2 | 8.8 | 6.6 |
| NM_008177 | Grpr | A_52_P646957 | 1.0 | 2.9 | 11.5 | 6.4 |
| NM_146120 | Gsn | A_51_P241465 | 1.0 | 1.2 | 8.4 | 12.5 |
| BC006955 | H2afy | A_52_P179238 | 1.0 | 1.7 | 3.6 | 7.6 |
| BC080756 | H2-K1 | A_51_P198434 | 1.0 | 1.1 | 3.1 | 10.3 |
| NM_173371 | H6pd | A_51_P321643 | 1.0 | 1.7 | 4.5 | 10.3 |
| NM_013500 | Hapln1 | A_51_P101787 | 1.0 | 26.1 | 185.9 | 10.7 |
| NM_178255 | Hapln3 | A_51_P244213 | 1.0 | 1.8 | 3.3 | 9.1 |
| NM_008216 | Has2 | A_51_P213359 | 1.0 | 2.1 | 3.1 | 9.2 |
| NM_010407 | Hck | A_51_P499918 | 1.0 | 9.7 | 34.5 | 8.3 |
| NM_133808 | Hdlbp | A_51_P416585 | 1.0 | 1.3 | 3.5 | 12.6 |
| NM_019487 | Hebp2 | A_51_P159612 | 1.0 | 1.7 | 3.4 | 9.0 |
| NM_172655 | Hecw2 | A_51_P406306 | 1.0 | 2.3 | 3.8 | 7.3 |
| NM_145070 | Hip1r | A_51_P440460 | 1.3 | 1.0 | 5.1 | 6.2 |
| NM_010437 | Hivep2 | A_51_P443754 | 1.0 | 2.2 | 5.3 | 6.0 |
| AK038070 | Hivep3 | A_52_P1131985 | 1.0 | 2.2 | 41.5 | 6.6 |
| NM_017370 | Hp | A_51_P235945 | 1.0 | 6.2 | 4.9 | 6.5 |
| NM_008281 | Hpn | A_51_P434483 | 1.0 | 1.3 | 3.3 | 8.2 |
| NM_010473 | Hrc | A_51_P267751 | 1.0 | 2.3 | 22.0 | 7.1 |
| NM_178870 | Hs3st3a1 | A_51_P515120 | 1.0 | 1.7 | 4.3 | 8.3 |
| NM_018805 | Hs3st3b1 | A_51_P410715 | 1.0 | 2.4 | 12.5 | 9.2 |
| NM_013560 | Hspb1 | A_52_P404533 | 1.0 | 1.3 | 3.5 | 7.8 |
| NM_019564 | Htra1 | A_51_P225224 | 1.0 | 3.6 | 8.6 | 9.9 |
| NM_030127 | Htra3 | A_51_P106538 | 1.0 | 2.4 | 4.9 | 9.5 |
| NM_026601 | Hyi | A_51_P377579 | 1.0 | 1.9 | 4.0 | 12.0 |
| NM_146042 | Ibrdc2 | A_52_P354752 | 1.0 | 1.6 | 3.9 | 7.9 |
| NM_053088 | Ifitm5 | A_52_P273812 | 1.0 | 1.0 | 9.9 | 6.6 |
| NM_010515 | Igf2r | A_51_P125467 | 1.0 | 1.9 | 4.1 | 13.1 |
| XM_887155 | Igsf10 | A_51_P155843 | 1.0 | 3.3 | 5.4 | 10.8 |
| AK090147 | Ihh | A_52_P403398 | 1.0 | 6.9 | 27.4 | 8.9 |
| NM_008349 | Il10rb | A_51_P406454 | 1.0 | 1.4 | 3.0 | 9.3 |
| NM_010551 | Il16 | A_51_P372702 | 1.0 | 1.1 | 4.1 | 8.3 |
| NM_145837 | Il17d | A_51_P154513 | 1.0 | 1.2 | 7.1 | 10.1 |
| NM_025454 | Ing5 | A_51_P110173 | 1.0 | 1.0 | 3.1 | 6.9 |
| AK166886 | Iqsec1 | A_52_P194289 | 1.0 | 1.6 | 3.0 | 8.6 |
| AK136875 | Irs1 | A_52_P175242 | 1.0 | 1.6 | 5.3 | 13.4 |
| NM_012043 | Islr | A_51_P140742 | 1.0 | 1.3 | 3.5 | 12.4 |
| AK040830 | Itga1 | A_51_P268944 | 1.0 | 1.6 | 3.7 | 6.2 |
| NM_176922 | Itga11 | A_51_P417720 | 1.0 | 2.2 | 4.5 | 10.4 |
| NM_133721 | Itga9 | A_51_P382970 | 1.0 | 1.4 | 3.2 | 10.4 |
| NM_001005608 | Itgb4 | A_51_P408382 | 1.0 | 1.5 | 3.7 | 7.9 |
| NM_019923 | Itpr2 | A_51_P500051 | 1.0 | 2.8 | 11.6 | 10.9 |
| J00623 | J00623 | A_52_P936271 | 1.0 | 3.8 | 4.5 | 8.5 |
| NM_177049 | Jph4 | A_51_P425352 | 1.0 | 1.3 | 3.1 | 10.4 |
| NM_178929 | Kazald1 | A_51_P493522 | 1.0 | 3.0 | 7.3 | 8.9 |
| BC080858 | Kbtbd11 | A_52_P570240 | 1.0 | 1.0 | 3.2 | 10.5 |
| NM_010597 | Kcnab1 | A_52_P568895 | 1.0 | 3.7 | 3.6 | 6.7 |
| NM_019789 | Kcnip3 | A_52_P185255 | 1.0 | 1.5 | 3.6 | 7.4 |
| NM_001033525 | Kcnk6 | A_51_P315890 | 1.0 | 1.2 | 7.1 | 7.9 |
| NR_001461 | Kcnq1ot1 | A_52_P619388 | 1.0 | 1.3 | 3.2 | 10.8 |
| NM_177715 | Kctd12 | A_51_P371912 | 1.0 | 1.4 | 5.3 | 7.9 |
| NM_134090 | Kdelr3 | A_52_P473966 | 1.0 | 2.3 | 5.7 | 10.3 |
| NM_021366 | Klf13 | A_51_P341169 | 1.0 | 1.2 | 4.0 | 8.5 |
| NM_023184 | Klf15 | A_52_P64383 | 1.0 | 1.8 | 4.6 | 8.4 |
| NM_008452 | Klf2 | A_51_P144264 | 1.0 | 6.4 | 31.2 | 11.6 |
| NM_010637 | Klf4 | A_51_P213544 | 1.0 | 3.1 | 19.5 | 10.6 |
| NM_026167 | Klhl13 | A_51_P521304 | 1.0 | 2.6 | 3.7 | 11.7 |
| NM_001033352 | Klhl21 | A_51_P477833 | 1.0 | 1.5 | 3.0 | 10.0 |
| NM_008940 | Klk8 | A_52_P216672 | 1.0 | 1.7 | 4.3 | 9.1 |
| NM_010681 | Lama4 | A_51_P212420 | 1.0 | 1.2 | 3.9 | 9.4 |
| NM_008483 | Lamb2 | A_51_P248044 | 1.0 | 1.4 | 3.4 | 10.3 |
| NM_029999 | Lbh | A_52_P539434 | 1.0 | 1.3 | 3.7 | 6.5 |
| NM_008879 | Lcp1 | A_52_P56751 | 1.3 | 1.0 | 4.4 | 8.3 |
| NM_010696 | Lcp2 | A_51_P352875 | 1.0 | 3.3 | 7.3 | 6.6 |
| BC053041 | Ldlr | A_52_P427024 | 1.1 | 1.0 | 3.3 | 12.0 |
| NM_010701 | Lect1 | A_51_P444645 | 1.0 | 1.2 | 6.9 | 7.4 |
| AK083328 | Lef1 | A_51_P501145 | 1.0 | 2.0 | 5.3 | 11.5 |
| NM_019782 | Lepre1 | A_51_P346923 | 1.0 | 1.3 | 4.5 | 8.2 |
| NM_010705 | Lgals3 | A_51_P101929 | 1.0 | 3.1 | 34.2 | 9.2 |
| AK122570 | Lgi2 | A_52_P446364 | 1.0 | 1.8 | 9.2 | 9.2 |
| NM_011175 | Lgmn | A_51_P110576 | 1.0 | 1.7 | 3.4 | 10.6 |
| NM_144862 | Lims2 | A_51_P151862 | 1.0 | 2.0 | 34.1 | 7.9 |
| AK011495 | Lman1 | A_52_P10781 | 1.0 | 1.3 | 4.0 | 10.3 |
| NM_144799 | Lmcd1 | A_51_P405912 | 1.0 | 2.8 | 3.9 | 10.2 |
| NM_019390 | Lmna | A_51_P313566 | 1.0 | 2.1 | 6.5 | 11.5 |
| DV040849 | Lmo7 | A_52_P68755 | 1.0 | 3.6 | 18.3 | 9.0 |
| NM_008508 | Lor | A_51_P495269 | 1.0 | 3.0 | 5.7 | 11.1 |
| NM_010728 | Lox | A_51_P300493 | 1.0 | 2.7 | 8.0 | 7.2 |
| NM_013586 | Loxl3 | A_52_P219233 | 1.0 | 1.7 | 3.7 | 7.6 |
| NM_172668 | Lrp4 | A_52_P635078 | 1.0 | 1.4 | 3.1 | 9.0 |
| BC030317 | Lrrc17 | A_51_P301930 | 1.0 | 2.6 | 3.0 | 13.0 |
| NM_145152 | Lrrc3 | A_51_P354609 | 1.0 | 1.3 | 3.4 | 6.7 |
| NM_010733 | Lrrn3 | A_51_P392303 | 1.0 | 1.9 | 7.4 | 9.3 |
| NM_181470 | Ltv1 | A_51_P197816 | 1.0 | 1.3 | 3.0 | 6.9 |
| NM_008524 | Lum | A_51_P167527 | 1.0 | 13.9 | 62.2 | 12.1 |
| NM_178705 | Luzp2 | A_52_P161455 | 1.0 | 1.4 | 4.0 | 6.2 |
| NM_010747 | Lyn | A_51_P345422 | 1.0 | 1.8 | 7.7 | 8.1 |
| BC099511 | Lypd2 | A_51_P136521 | 1.0 | 1.6 | 3.8 | 8.3 |
| NM_001025577 | Maf | A_51_P405167 | 1.0 | 3.4 | 6.1 | 11.5 |
| NM_010658 | Mafb | A_52_P495869 | 1.0 | 3.9 | 4.7 | 9.7 |
| NM_145532 | Mall | A_51_P221441 | 1.2 | 1.0 | 4.8 | 8.5 |
| NM_008580 | Map3k5 | A_52_P656714 | 1.0 | 2.3 | 7.0 | 7.3 |
| NM_010769 | Matn1 | A_52_P598420 | 1.0 | 6.7 | 25.5 | 10.0 |
| NM_016762 | Matn2 | A_51_P484111 | 1.0 | 2.5 | 20.4 | 8.4 |
| NM_010770 | Matn3 | A_52_P297093 | 1.0 | 5.6 | 30.2 | 9.4 |
| NM_013592 | Matn4 | A_51_P264934 | 1.0 | 2.0 | 4.4 | 13.2 |
| AK088871 | Mbnl1 | A_52_P228437 | 1.0 | 2.1 | 7.2 | 8.9 |
| NM_020007 | Mbnl1 | A_51_P428345 | 1.0 | 2.0 | 4.5 | 12.3 |
| NM_001025245 | Mbp | A_52_P604195 | 1.0 | 3.1 | 7.1 | 8.4 |
| NM_026656 | Mcoln2 | A_51_P434301 | 1.0 | 2.9 | 16.2 | 6.9 |
| NM_025282 | Mef2c | A_51_P135802 | 1.0 | 1.7 | 5.5 | 10.5 |
| NM_144797 | Metrnl | A_52_P355084 | 1.0 | 2.5 | 7.5 | 10.1 |
| NM_008594 | Mfge8 | A_51_P255682 | 1.0 | 1.6 | 4.7 | 13.8 |
| NM_013900 | Mfi2 | A_51_P324351 | 1.0 | 2.0 | 21.5 | 9.9 |
| AK036567 | Mgat5 | A_52_P24696 | 1.0 | 1.1 | 3.5 | 7.1 |
| NM_011844 | Mgll | A_52_P609778 | 1.0 | 2.2 | 10.1 | 11.4 |
| NM_008597 | Mgp | A_51_P426270 | 1.0 | 5.0 | 26.9 | 12.4 |
| NM_174995 | Mgst2 | A_51_P150120 | 1.0 | 4.3 | 13.9 | 9.8 |
| NM_019394 | Mia1 | A_52_P415168 | 1.0 | 7.7 | 15.4 | 12.9 |
| NM_011845 | Mid2 | A_52_P368473 | 1.0 | 1.8 | 5.3 | 8.7 |
| AK048543 | Mmp16 | A_52_P975 | 1.0 | 1.0 | 3.2 | 8.5 |
| NM_013599 | Mmp9 | A_51_P401797 | 1.0 | 5.3 | 24.6 | 7.5 |
| NM_029413 | Morc4 | A_52_P147816 | 1.0 | 1.9 | 3.8 | 8.6 |
| NM_008619 | Mov10 | A_51_P169495 | 1.0 | 1.7 | 3.2 | 10.6 |
| NM_021509 | Moxd1 | A_51_P493987 | 1.0 | 6.3 | 27.3 | 10.6 |
| NM_177092 | Msrb3 | A_52_P121491 | 1.0 | 1.1 | 4.5 | 9.0 |
| AK018185 | Mtap1a | A_52_P593278 | 1.0 | 1.5 | 3.6 | 7.5 |
| NM_008638 | Mthfd2 | A_52_P184149 | 1.0 | 1.1 | 3.5 | 9.5 |
| NM_178902 | Mtus1 | A_51_P341801 | 1.0 | 1.9 | 3.1 | 8.5 |
| NM_016749 | Mybph | A_51_P137121 | 1.0 | 2.4 | 4.1 | 6.1 |
| XM_354614 | Myh3 | A_51_P167668 | 1.0 | 3.2 | 8.6 | 7.9 |
| NM_080728 | Myh7 | A_51_P143162 | 1.3 | 1.0 | 5.9 | 6.2 |
| NM_021285 | Myl1 | A_51_P416858 | 1.0 | 1.0 | 4.3 | 8.6 |
| NM_010859 | Myl3 | A_51_P177210 | 1.0 | 2.0 | 4.4 | 7.5 |
| NM_010858 | Myl4 | A_52_P544523 | 1.0 | 1.8 | 4.3 | 6.7 |
| NM_008664 | Myom2 | A_51_P431785 | 1.0 | 1.6 | 10.6 | 7.3 |
| NM_008669 | Naga | A_51_P117130 | 1.0 | 1.3 | 3.2 | 8.6 |
| X15052 | Ncam1 | A_52_P613255 | 1.0 | 2.3 | 6.0 | 10.2 |
| NM_010883 | Ndph | A_51_P429903 | 1.0 | 2.5 | 3.7 | 6.1 |
| NM_025424 | Nenf | A_51_P126987 | 1.0 | 1.7 | 3.2 | 12.4 |
| AK004524 | Neurl2 | A_51_P418935 | 1.0 | 2.1 | 5.6 | 9.5 |
| NM_010906 | Nfix | A_51_P478598 | 1.0 | 2.0 | 4.9 | 9.9 |
| NM_019867 | Ngef | A_51_P170823 | 1.0 | 1.3 | 4.9 | 7.8 |
| NM_029342 | Nhej1 | A_52_P128964 | 1.0 | 2.4 | 6.0 | 10.5 |
| NM_008697 | Nin | A_52_P597696 | 1.0 | 1.4 | 3.1 | 9.2 |
| NM_013610 | Ninj1 | A_51_P295046 | 1.0 | 1.9 | 3.4 | 10.2 |
| NM_133800 | Nol12 | A_51_P189916 | 1.0 | 1.4 | 3.0 | 11.8 |
| NM_030152 | Nol3 | A_51_P273170 | 1.0 | 1.3 | 3.0 | 7.1 |
| NM_153057 | Nomo1 | A_51_P105520 | 1.0 | 1.2 | 5.0 | 11.9 |
| NM_173788 | Npr2 | A_51_P518940 | 1.0 | 1.8 | 3.6 | 10.8 |
| AK171439 | Nr3c1 | A_52_P138090 | 1.0 | 1.2 | 3.5 | 8.3 |
| NM_144847 | Nrbp2 | A_51_P296036 | 1.0 | 1.3 | 6.5 | 8.9 |
| NM_022029 | Nrgn | A_51_P171832 | 1.0 | 2.2 | 5.4 | 8.1 |
| NM_008738 | Nrtn | A_52_P463968 | 1.0 | 1.6 | 5.0 | 9.6 |
| NM_020253 | Nrxn2 | A_51_P431831 | 1.5 | 1.0 | 4.9 | 6.2 |
| NM_011851 | Nt5e | A_52_P574668 | 1.0 | 1.2 | 7.3 | 6.4 |
| NM_018815 | Nup210 | A_51_P420037 | 1.0 | 1.0 | 7.6 | 8.4 |
| NM_019738 | Nupr1 | A_51_P519251 | 1.0 | 1.2 | 27.0 | 7.1 |
| NM_008760 | Ogn | A_51_P295085 | 1.0 | 3.5 | 10.8 | 9.4 |
| NM_017375 | Ostf1 | A_51_P277345 | 1.0 | 1.3 | 3.9 | 9.6 |
| NM_024188 | Oxct1 | A_51_P107326 | 1.0 | 1.5 | 3.0 | 13.8 |
| NM_008773 | P2ry2 | A_52_P587738 | 1.0 | 1.4 | 8.5 | 6.5 |
| NM_008778 | Pak3 | A_51_P218345 | 1.0 | 2.5 | 9.4 | 8.8 |
| NM_172454 | Panx3 | A_52_P27190 | 1.0 | 11.0 | 298.1 | 7.8 |
| NM_008788 | Pcolce | A_51_P371942 | 1.0 | 1.6 | 3.1 | 12.9 |
| NM_029620 | Pcolce2 | A_51_P245789 | 1.0 | 1.7 | 6.0 | 12.4 |
| XM_355911 | Pcsk6 | A_52_P376574 | 1.0 | 2.4 | 4.6 | 11.5 |
| NM_001039376 | Pde4dip | A_51_P489488 | 1.0 | 4.5 | 57.2 | 9.8 |
| NM_008803 | Pde8a | A_52_P391000 | 1.7 | 1.0 | 4.9 | 7.2 |
| NM_008808 | Pdgfa | A_51_P370090 | 1.0 | 2.4 | 26.8 | 11.8 |
| NM_026840 | Pdgfrl | A_51_P466229 | 1.0 | 2.2 | 3.7 | 10.7 |
| NM_001002927 | Penk1 | A_51_P102987 | 1.0 | 4.6 | 7.0 | 12.9 |
| NM_022032 | Perp | A_51_P317941 | 1.9 | 1.0 | 6.2 | 7.5 |
| NM_011077 | Phex | A_51_P468249 | 1.0 | 4.6 | 5.7 | 6.6 |
| NM_009434 | Phlda2 | A_51_P450623 | 1.0 | 5.2 | 18.5 | 12.4 |
| NM_011085 | Pik3r1 | A_52_P407239 | 1.0 | 2.0 | 6.6 | 6.4 |
| AK172137 | Pitpnc1 | A_52_P82023 | 1.0 | 1.0 | 3.4 | 8.7 |
| NM_134102 | Pla1a | A_51_P381618 | 1.0 | 2.0 | 10.8 | 7.3 |
| NM_011110 | Pla2g5 | A_52_P94983 | 1.0 | 2.2 | 14.6 | 6.6 |
| NM_207229 | Plac9 | A_51_P215627 | 1.0 | 4.7 | 5.8 | 8.8 |
| BC065150 | Plagl1 | A_52_P532456 | 1.0 | 1.4 | 3.4 | 14.3 |
| NM_019676 | Plcd1 | A_51_P239766 | 1.0 | 2.1 | 7.5 | 11.3 |
| NM_172285 | Plcg2 | A_51_P279163 | 1.0 | 2.3 | 9.4 | 9.6 |
| NM_013880 | Plcl2 | A_51_P219444 | 1.0 | 2.1 | 3.4 | 10.7 |
| BC025841 | Plekha6 | A_51_P310850 | 1.2 | 1.0 | 9.1 | 6.1 |
| NM_011962 | Plod3 | A_51_P372172 | 1.0 | 1.2 | 3.6 | 11.4 |
| NM_011125 | Pltp | A_51_P226655 | 1.0 | 2.6 | 18.9 | 6.9 |
| AK011113 | Plxnc1 | A_52_P507498 | 1.0 | 1.4 | 3.4 | 6.9 |
| NM_080463 | Pofut1 | A_52_P665833 | 1.0 | 1.5 | 3.3 | 8.4 |
| NM_030262 | Pofut2 | A_52_P296682 | 1.0 | 1.4 | 3.6 | 11.2 |
| NM_145610 | Ppan | A_51_P189985 | 1.0 | 1.1 | 3.2 | 12.5 |
| NM_133249 | Ppargc1b | A_51_P294891 | 1.0 | 2.2 | 4.2 | 7.8 |
| NM_177167 | Ppm1e | A_52_P607683 | 1.7 | 1.0 | 11.2 | 7.4 |
| NM_028392 | Ppp2r2b | A_51_P220278 | 1.0 | 5.0 | 11.6 | 7.7 |
| NM_054077 | Prelp | A_52_P486260 | 1.0 | 2.6 | 18.0 | 11.9 |
| NM_028444 | Prkcdbp | A_51_P180974 | 1.0 | 1.5 | 4.7 | 10.3 |
| NM_008926 | Prkg2 | A_51_P273433 | 1.0 | 4.1 | 22.8 | 8.7 |
| NM_013640 | Psmb10 | A_52_P570266 | 1.0 | 1.6 | 3.2 | 11.0 |
| NM_008969 | Ptgs1 | A_51_P279100 | 1.0 | 2.4 | 3.8 | 9.3 |
| NM_008970 | Pthlh | A_51_P129360 | 1.0 | 5.1 | 4.1 | 8.2 |
| NM_011199 | Pthr1 | A_51_P327874 | 1.0 | 2.0 | 8.1 | 12.4 |
| NM_011204 | Ptpn13 | A_51_P455208 | 1.0 | 1.6 | 3.0 | 9.6 |
| NM_007955 | Ptprv | A_51_P150653 | 1.0 | 1.9 | 12.8 | 6.8 |
| NM_008986 | Ptrf | A_51_P237752 | 1.0 | 3.0 | 10.7 | 11.9 |
| NM_144795 | Pycr1 | A_51_P503896 | 1.0 | 1.2 | 3.6 | 9.2 |
| BC052065 | Rap1gap | A_51_P289862 | 1.0 | 1.3 | 3.5 | 10.3 |
| NM_009025 | Rasa3 | A_51_P178063 | 1.0 | 1.1 | 3.2 | 10.5 |
| BC051474 | Rasgrp2 | A_52_P420734 | 1.0 | 1.0 | 3.2 | 8.5 |
| NM_009036 | Rbpsuhl | A_52_P574759 | 1.0 | 1.3 | 3.2 | 7.4 |
| NM_026555 | Rcn3 | A_51_P398683 | 1.0 | 1.8 | 5.9 | 13.9 |
| NM_178593 | Rcsd1 | A_51_P371091 | 1.0 | 2.2 | 5.7 | 10.1 |
| NM_181988 | Rerg | A_51_P305508 | 1.0 | 1.1 | 12.2 | 6.8 |
| NM_021375 | Rhbg | A_51_P400366 | 1.0 | 1.9 | 9.9 | 7.1 |
| NM_007483 | Rhob | A_52_P89567 | 1.0 | 1.1 | 3.1 | 8.0 |
| NM_020599 | Rlbp1 | A_51_P211793 | 1.0 | 1.5 | 5.1 | 6.9 |
| NM_021472 | Rnase4 | A_51_P237383 | 1.0 | 1.7 | 3.0 | 9.3 |
| NM_019924 | Rps6ka4 | A_51_P482633 | 1.0 | 1.8 | 5.8 | 8.9 |
| XM_622097 | Rrbp1 | A_51_P345663 | 1.0 | 1.3 | 4.0 | 12.4 |
| NM_009820 | Runx2 | A_51_P230942 | 1.0 | 13.1 | 10.8 | 11.8 |
| NM_019732 | Runx3 | A_52_P666059 | 1.0 | 3.1 | 5.9 | 9.0 |
| NM_011309 | S100a1 | A_51_P172475 | 1.0 | 1.8 | 8.1 | 9.5 |
| NM_009112 | S100a10 | A_51_P342871 | 1.0 | 1.7 | 3.6 | 10.4 |
| NM_011311 | S100a4 | A_51_P105078 | 1.0 | 2.5 | 5.6 | 8.8 |
| NM_011313 | S100a6 | A_51_P281089 | 1.0 | 5.1 | 6.9 | 9.5 |
| NM_009115 | S100b | A_51_P208083 | 1.0 | 4.7 | 134.7 | 9.6 |
| NM_009117 | Saa1 | A_52_P318673 | 1.0 | 4.0 | 3.1 | 9.7 |
| NM_011314 | Saa2 | A_51_P166886 | 1.0 | 6.9 | 5.3 | 9.9 |
| NM_138665 | Sardh | A_51_P492676 | 1.0 | 2.0 | 4.7 | 7.5 |
| NM_016741 | Scarb1 | A_51_P255817 | 1.0 | 1.2 | 3.7 | 10.9 |
| NM_009132 | Scin | A_51_P335460 | 1.0 | 12.1 | 57.7 | 10.1 |
| NM_009136 | Scrg1 | A_51_P258690 | 1.0 | 2.6 | 4.7 | 10.1 |
| NM_011328 | Sct | A_51_P234359 | 1.0 | 2.0 | 4.4 | 9.3 |
| NM_020052 | Scube2 | A_51_P192397 | 1.0 | 1.7 | 4.8 | 6.7 |
| NM_008304 | Sdc2 | A_51_P292403 | 1.0 | 2.6 | 3.9 | 9.0 |
| NM_011521 | Sdc4 | A_52_P93467 | 1.0 | 1.8 | 4.2 | 9.7 |
| AK129379 | Sdk2 | A_52_P527977 | 1.0 | 2.3 | 14.3 | 7.9 |
| NM_144520 | Sec14l2 | A_52_P488427 | 1.0 | 1.4 | 3.7 | 9.5 |
| NM_016906 | Sec61a1 | A_52_P489729 | 1.0 | 1.3 | 3.2 | 13.4 |
| NM_053267 | Selm | A_52_P357133 | 1.0 | 2.3 | 3.5 | 12.4 |
| NM_172588 | Serinc5 | A_51_P128336 | 1.0 | 3.1 | 28.5 | 10.6 |
| NM_009825 | Serpinh1 | A_51_P287069 | 1.0 | 1.5 | 5.1 | 14.1 |
| NM_013781 | Sh2d3c | A_51_P464761 | 1.0 | 1.2 | 3.2 | 8.4 |
| NM_172628 | Sh3tc2 | A_51_P202596 | 1.0 | 1.8 | 7.0 | 8.8 |
| NM_021882 | Si | A_51_P208145 | 1.0 | 1.2 | 3.8 | 6.9 |
| NM_172257 | Sidt2 | A_51_P385961 | 1.0 | 1.1 | 3.4 | 11.4 |
| NM_030749 | Sil1 | A_51_P438200 | 1.0 | 2.4 | 4.1 | 11.5 |
| NM_009195 | Slc12a4 | A_51_P251256 | 1.0 | 1.9 | 3.7 | 11.1 |
| NM_172892 | Slc13a4 | A_51_P514319 | 1.0 | 1.7 | 3.3 | 7.9 |
| NM_030696 | Slc16a3 | A_51_P144090 | 1.2 | 1.0 | 4.9 | 12.9 |
| NM_134038 | Slc16a6 | A_51_P432180 | 1.0 | 1.1 | 3.1 | 8.3 |
| NM_009199 | Slc1a1 | A_51_P110759 | 1.0 | 1.2 | 4.4 | 6.7 |
| NM_018861 | Slc1a4 | A_51_P371311 | 1.3 | 1.0 | 4.5 | 6.0 |
| NM_007885 | Slc26a2 | A_52_P83905 | 1.0 | 1.0 | 3.1 | 7.9 |
| NM_001033633 | Slc2a13 | A_52_P607255 | 1.0 | 1.1 | 5.5 | 8.2 |
| NM_144902 | Slc35a3 | A_52_P413855 | 1.0 | 2.2 | 6.8 | 9.1 |
| NM_211358 | Slc35c1 | A_51_P443723 | 1.0 | 1.3 | 3.7 | 10.9 |
| NM_177732 | Slc35d1 | A_51_P266964 | 1.0 | 2.1 | 16.6 | 10.6 |
| NM_153142 | Slc35e4 | A_51_P268934 | 1.0 | 1.8 | 3.8 | 9.3 |
| NM_178675 | Slc35f1 | A_52_P26299 | 1.0 | 1.4 | 4.7 | 8.7 |
| NM_023805 | Slc38a3 | A_51_P139030 | 1.0 | 1.3 | 4.1 | 8.0 |
| NM_172479 | Slc38a5 | A_51_P400269 | 1.0 | 1.6 | 3.2 | 6.9 |
| NM_144808 | Slc39a14 | A_51_P282983 | 1.0 | 2.1 | 6.5 | 9.6 |
| NM_016917 | Slc40a1 | A_51_P389988 | 1.0 | 1.2 | 4.3 | 9.4 |
| BC052435 | Slc8a3 | A_52_P7610 | 1.0 | 2.3 | 4.8 | 6.7 |
| AK077026 | Slc9a2 | A_51_P397983 | 1.0 | 4.6 | 59.6 | 8.3 |
| AK019479 | Slit3 | A_51_P355765 | 1.0 | 1.7 | 3.1 | 10.4 |
| NM_025540 | Sln | A_52_P413395 | 1.0 | 1.9 | 5.1 | 6.2 |
| NM_011414 | Slpi | A_52_P472324 | 1.0 | 2.4 | 8.5 | 9.0 |
| NM_022315 | Smoc2 | A_52_P646762 | 1.0 | 3.3 | 6.6 | 8.3 |
| NM_021491 | Smpd3 | A_52_P478025 | 1.0 | 3.7 | 75.0 | 9.7 |
| NM_009228 | Snta1 | A_51_P339232 | 1.0 | 1.2 | 4.9 | 8.2 |
| NM_009230 | Soat1 | A_51_P391754 | 1.0 | 1.5 | 3.1 | 10.3 |
| NM_007706 | Socs2 | A_51_P107362 | 1.0 | 3.1 | 3.8 | 10.5 |
| NM_009166 | Sorbs1 | A_51_P253117 | 1.0 | 4.0 | 12.3 | 12.5 |
| BC039163 | Sorbs2 | A_52_P403157 | 1.0 | 1.0 | 4.6 | 8.8 |
| NM_019972 | Sort1 | A_52_P590396 | 1.0 | 1.4 | 3.1 | 10.2 |
| NM_011447 | Sox8 | A_52_P269672 | 1.0 | 1.6 | 3.8 | 8.6 |
| NM_130458 | Sp7 | A_52_P236709 | 1.0 | 2.8 | 8.1 | 8.3 |
| NM_009242 | Sparc | A_52_P101852 | 1.0 | 1.6 | 3.8 | 12.7 |
| NM_052994 | Spock2 | A_52_P221756 | 1.0 | 1.6 | 3.2 | 6.4 |
| NM_009263 | Spp1 | A_51_P358765 | 1.4 | 1.0 | 4.5 | 8.1 |
| NM_001033277 | Spryd3 | A_51_P470228 | 1.0 | 1.4 | 3.3 | 10.3 |
| NM_009219 | Sstr4 | A_52_P145628 | 1.0 | 5.9 | 6.6 | 7.3 |
| NM_009177 | St3gal1 | A_51_P301804 | 1.0 | 1.5 | 5.1 | 9.2 |
| NM_145838 | St8sia6 | A_51_P471791 | 1.0 | 2.0 | 7.9 | 6.4 |
| NM_027399 | Steap1 | A_51_P484158 | 1.0 | 1.2 | 4.0 | 8.9 |
| NM_009288 | Stk10 | A_51_P192130 | 1.0 | 1.4 | 3.3 | 8.8 |
| NM_009291 | Stra6 | A_51_P471177 | 1.0 | 2.2 | 3.5 | 8.2 |
| NM_029659 | Styxl1 | A_51_P227042 | 1.0 | 1.5 | 3.1 | 6.6 |
| NM_022814 | Svep1 | A_51_P394814 | 1.0 | 3.8 | 5.1 | 9.7 |
| NM_177089 | Tacc1 | A_51_P233982 | 1.0 | 1.3 | 3.6 | 9.3 |
| NM_178598 | Tagln2 | A_52_P35240 | 1.0 | 2.0 | 3.5 | 11.6 |
| NM_025289 | Tbrg1 | A_51_P186531 | 1.0 | 1.5 | 3.8 | 11.5 |
| NM_001037878 | Tcf25 | A_52_P319581 | 1.0 | 1.5 | 3.9 | 11.3 |
| NM_009331 | Tcf7 | A_52_P244702 | 1.8 | 1.0 | 9.8 | 6.6 |
| NM_011552 | Tcof1 | A_51_P160284 | 1.0 | 1.4 | 3.5 | 9.8 |
| D87966 | Tead4 | A_51_P179041 | 1.1 | 1.0 | 3.9 | 6.2 |
| NM_153533 | Tenc1 | A_51_P395473 | 1.0 | 3.0 | 5.9 | 9.8 |
| NM_021344 | Tesc | A_52_P69955 | 1.0 | 3.7 | 10.2 | 7.0 |
| NM_011577 | Tgfb1 | A_51_P390715 | 1.0 | 1.1 | 3.5 | 6.7 |
| NM_009373 | Tgm2 | A_52_P220879 | 1.0 | 4.4 | 45.5 | 10.7 |
| NM_009444 | Tgoln2 | A_52_P375764 | 1.0 | 1.3 | 3.6 | 8.9 |
| NM_009378 | Thbd | A_51_P291417 | 1.0 | 7.2 | 5.2 | 8.1 |
| NM_011582 | Thbs4 | A_51_P353221 | 1.0 | 1.4 | 40.1 | 6.4 |
| AK158826 | Thrb | A_52_P532559 | 1.0 | 1.3 | 6.4 | 6.2 |
| NM_009382 | Thy1 | A_52_P479249 | 1.0 | 1.9 | 30.1 | 7.7 |
| NM_011878 | Tiam2 | A_51_P256066 | 1.0 | 1.9 | 3.6 | 12.3 |
| NM_011593 | Timp1 | A_52_P87713 | 1.0 | 1.9 | 5.6 | 11.4 |
| NM_026708 | Tlcd1 | A_51_P312275 | 1.0 | 1.5 | 4.3 | 9.0 |
| NM_030682 | Tlr1 | A_51_P495581 | 1.0 | 1.7 | 3.2 | 8.5 |
| BC049960 | Tm7sf3 | A_52_P405206 | 1.0 | 1.7 | 3.7 | 9.5 |
| NM_025360 | Tmed3 | A_51_P284244 | 1.0 | 1.5 | 3.5 | 13.3 |
| AK009669 | Tmem158 | A_51_P104012 | 1.0 | 3.0 | 3.0 | 12.0 |
| AK052589 | Tmem16a | A_51_P168894 | 1.0 | 1.7 | 7.6 | 6.2 |
| NM_175507 | Tmem20 | A_51_P423608 | 1.0 | -1.0 | 6.0 | 8.3 |
| NM_133718 | Tmem30a | A_52_P576778 | 1.0 | 1.8 | 3.3 | 10.0 |
| NM_019432 | Tmem37 | A_51_P401987 | 1.0 | 2.7 | 3.8 | 9.8 |
| NM_019631 | Tmem45a | A_51_P288876 | 1.0 | 1.5 | 3.1 | 11.0 |
| NM_146260 | Tmie | A_52_P89335 | 1.0 | 1.6 | 3.2 | 7.2 |
| NM_011607 | Tnc | A_51_P291438 | 1.0 | 6.5 | 9.2 | 8.0 |
| NM_013749 | Tnfrsf12a | A_51_P131408 | 1.0 | 2.5 | 3.2 | 8.7 |
| NM_009400 | Tnfrsf18 | A_51_P469401 | 1.0 | 2.1 | 4.1 | 6.4 |
| NM_013869 | Tnfrsf19 | A_51_P399305 | 1.0 | 1.7 | 4.6 | 6.7 |
| NM_178589 | Tnfrsf21 | A_51_P168762 | 1.1 | 1.0 | 3.6 | 7.5 |
| NM_009394 | Tnnc2 | A_51_P353232 | 1.0 | 2.3 | 3.8 | 8.5 |
| NM_021467 | Tnni1 | A_52_P657360 | 1.0 | 2.9 | 6.3 | 8.7 |
| NM_009405 | Tnni2 | A_51_P355122 | 1.0 | 3.1 | 7.3 | 9.0 |
| NM_011620 | Tnnt3 | A_52_P622418 | 1.0 | 1.3 | 3.1 | 6.2 |
| NM_145853 | Tpcn1 | A_52_P476560 | 1.0 | 1.5 | 6.1 | 10.5 |
| NM_172570 | Trim47 | A_51_P437176 | 1.0 | 5.7 | 26.6 | 10.7 |
| NM_175130 | Trpm4 | A_52_P303100 | 1.0 | 1.5 | 4.2 | 10.4 |
| NM_020277 | Trpm5 | A_51_P398423 | 1.0 | 1.9 | 6.1 | 8.2 |
| NM_022017 | Trpv4 | A_51_P269203 | 1.0 | 5.9 | 38.5 | 11.7 |
| NM_183180 | Tspan18 | A_52_P274496 | 1.0 | 2.2 | 9.2 | 10.0 |
| NM_020286 | Tspan32 | A_52_P474775 | 1.0 | 1.5 | 10.2 | 6.5 |
| NM_053082 | Tspan4 | A_51_P509609 | 1.0 | 5.4 | 18.8 | 13.0 |
| NM_028639 | Ttc7 | A_51_P103209 | 1.0 | 2.0 | 5.7 | 8.7 |
| BC021404 | Ttll3 | A_51_P153079 | 1.0 | 3.2 | 7.5 | 8.7 |
| NM_009450 | Tubb2a | A_51_P490023 | 1.0 | 1.3 | 5.0 | 12.2 |
| NM_026473 | Tubb6 | A_51_P421140 | 1.0 | 3.0 | 7.7 | 11.4 |
| NM_134105 | Txndc11 | A_51_P413545 | 1.0 | 1.4 | 3.3 | 10.7 |
| NM_001033293 | Uap1l1 | A_51_P509669 | 1.0 | 1.6 | 3.3 | 8.5 |
| NM_023738 | Ube1l | A_51_P316816 | 1.0 | 2.2 | 3.0 | 10.4 |
| AK088515 | Usp15 | A_52_P475033 | 1.0 | 2.8 | 3.5 | 8.0 |
| XM_126772 | Usp36 | A_52_P627925 | 1.5 | 1.0 | 6.3 | 7.5 |
| BC022221 | Usp53 | A_51_P358423 | 1.0 | 1.7 | 3.3 | 7.1 |
| AK029782 | Ust | A_52_P582568 | 1.0 | 4.8 | 5.5 | 7.7 |
| NM_198166 | Uts2d | A_52_P420665 | 1.0 | 6.4 | 4.0 | 7.7 |
| BC024687 | Vangl1 | A_52_P449417 | 1.0 | 1.9 | 3.6 | 10.2 |
| NM_009504 | Vdr | A_52_P334562 | 1.0 | 2.2 | 18.2 | 7.0 |
| NM_178600 | Vkorc1 | A_51_P395555 | 1.0 | 1.5 | 3.2 | 13.3 |
| NM_018865 | Wisp1 | A_51_P220343 | 1.0 | 1.9 | 4.7 | 9.4 |
| NM_175638 | Wnk4 | A_51_P486217 | 1.0 | 1.1 | 3.1 | 9.1 |
| NM_009523 | Wnt4 | A_51_P130475 | 1.0 | 2.6 | 17.2 | 7.2 |
| NM_026553 | Yif1a | A_51_P175736 | 1.0 | 1.6 | 4.3 | 7.8 |
| AK038731 | Zbtb20 | A_52_P657844 | 1.0 | 1.1 | 6.1 | 8.9 |
| NM_145356 | Zbtb7c | A_52_P65237 | 1.0 | 1.7 | 5.0 | 6.3 |
| NM_199468 | Zcchc5 | A_51_P370640 | 1.0 | 1.6 | 3.4 | 7.9 |
| NM_009549 | Zfp185 | A_52_P384264 | 1.0 | 1.8 | 4.2 | 7.7 |
| NM_178723 | Zfp533 | A_51_P451275 | 1.0 | 1.2 | 4.5 | 7.1 |
| NM_133218 | Zfp704 | A_51_P399853 | 1.0 | 3.0 | 3.1 | 10.4 |
| NM_172700 | Zmpste24 | A_52_P420438 | 1.0 | 1.2 | 3.3 | 9.6 |
