# Supplementary material for: Global comparative transcriptome analysis of cartilage formation in vivo
Source: BMC Dev Biol. 2009 Mar 10;9:20. doi: 10.1186/1471-213X-9-20 (PMC2662817; doi:10.1186/1471-213X-9-20)
Supplement: Additional file 3 — Validation of microarray expression data by comparative literature analysis. The data provided represent the differential expression profiles for a list of 50 genes whose differential expression during chondrogenesis have been corroborated elsewhere in the scientific literature, and includes specific references to which the reader is directed for each gene. [file 1471-213X-9-20-S3.doc]

|  | **Relative Fold Difference:** | | |  |
| --- | --- | --- | --- | --- |
| **Gene** | **11.5dpc** | **12.5dpc** | **13.5dpc** | **Reference** |
| Agc1 | 1.0 | 4.8 | 30.0 | Kiani et al (2002). Cell Research 12:19-32 |
| Bmp6 | 1.0 | 1.5 | 9.6 | Solloway et al (1998). Developmental Genetics 22:321-339 |
| Bmp7 | 1.0 | 3.8 | 13.2 | Pathi et al (1999). Developmental Biology 209:239-253 |
| Bmp8a | 1.0 | 2.0 | 50.8 | DiLeone et al (1997). Genomics 40:196-198 |
| Cdh11 | 1.7 | 2.3 | 1.0 | Luo et al (2005). Developmental Dynamics 232(2):336-344 |
| Col2a1 Col6a1  Col6a2  Col6a3 Col9a1 Col9a2 Col9a3 Col11a1 Col11a2 Col14a1 | 1.0  1.0  1.0  1.0  1.0  1.0  1.0  1.0  1.0  1.0 | 1.6  1.4  1.0  2.7  1.3  1.8  1.5  4.4  4.0  4.0 | 5.8  1.7  2.1  15.3  4.1  6.1  7.0  21.3  33.9  11.1 | Kielty and Grant (2002). The Collagen Family: Structure, Assembly, and Organization in the Extracellular Matrix. In Royce PM, Steinmann B (eds): “Connective tissue and its heritable disorders” New York: Wiley-Liss, Inc., pp159-221 |
| Cspg4 | 1.0 | 3.7 | 15.9 | Fukushi et al (2003). Developmental Dynamics 228:143-148 |
| Ctgf | 1.0 | 13.0 | 29.9 | Takigawa et al (2003). Journal of Cell Physiology 194:256-266 |
| Cyr61 | 1.0 | 1.5 | 3.2 | O’Brien and Lau (1992). Cell Growth and Differentiation 3:645-654 |
| Dspg3 | 1.0 | 3.7 | 39.3 | Johnson et al (1999). Developmental Dynamics 216(4-5):499-510 |
| Fgf18  Fgfr3 | 1.0  1.0 | 1.4  1.2 | 3.7  2.7 | Liu et al (2007). Developmental Biology 302:80-91 |
| Foxa3 | 1.0 | 6.4 | 20.5 | Monaghan et al (1993). Development 119:567-578 |
| Foxc2 | 1.0 | 3.5 | 2.8 | Nifuji et al (2001). Journal of Bone and Mineral Research 16:1765-1771 |
| Gdf5  Gdf6 | 2.1  1.6 | 3.0  4.3 | 1.0  1.0 | Settle et al (2003). Developmental Biology 254:116-130 |
| Hoxa9 Hoxa10 Hoxa11 Hoxc6 Hoxc9 Hoxd9 Hoxd10 Hoxd11 | 2.4  5.1  2.0  2.3  1.0  5.5  8.5  7.2 | 1.0  3.1  1.6  1.0  1.1  2.4  5.0  3.1 | 1.0  1.0  1.0  1.2  1.1  1.0  1.0  1.0 | Nelson et al (1996). Development 122:1449-1466 |
| Mmp9 | 1.0 | 5.3 | 24.5 | Vu et al (1998). Cell 93:411-422 |
| Msx2 | 3.2 | 1.3 | 1.0 | Satokata et al (2000). Nature Genetics 24:391-395 |
| Ncad | 2.8 | 1.3 | 1.0 | Oberlender and Tuan (1994). Development 120:177-187 |
| Pdgfra | 3.4 | 2.5 | 1.0 | Ataliotis (2000). Mechanisms of Development 94:13-24 |
| Runx3 | 1.0 | 3.1 | 5.9 | Stricker et al (2002). Developmental Biology 245:95-108 |
| Sox8 Sox10 | 1.0  1.0 | 1.6  1.2 | 3.8  2.1 | Chimal-Monroy et al (2003). Developmental Biology 257(2):292-301 |
| Sox9 | 1.4 | 1.0 | 1.1 | Wright et al (1995). Nature Genetics 9:15-20 |
| Spp1 | 1.4 | 1.0 | 4.5 | Shibata et al (2002). Journal of Anatomy 200:309-320 |
| Stmn1 | 3.1 | 2.4 | 1.0 | Hummert et al (2001). Endocrine 15:93-101 |
| Tagln2 | 1.0 | 2.0 | 3.5 | Zhang et al (2002). Mechanisms of Development 115:161-166 |
| Tbx3 Tbx18 | 6.6  8.9 | 1.9  4.9 | 1.0  1.0 | King et al (2006). American Journal of Medical Genetics. Part A 140:1407-1413 |
| Tgfbi | 1.0 | 1.4 | 1.5 | Han et al (2008). Experimental and Molecular Medicine 40(4):453-460 |
| Vdr | 1.0 | 2.2 | 18.2 | Balmain et al (1993). Cellular and Molecular Biology (Noisy-le-grand) 39:339-350 |
| Wisp1 | 1.0 | 1.9 | 4.7 | French et al (2004). American Journal of Pathology 165:855-867 |
| Wnt4 | 1.0 | 2.6 | 17.2 | Church et al (2002). Journal of Cell Science 115:4809-4818 |
